# Early Detection and Investigation of Extracellular Vesicles Biomarkers in Breast Cancer

**DOI:** 10.3389/fmolb.2021.732900

**Published:** 2021-11-08

**Authors:** Erika Bandini, Tania Rossi, Emanuela Scarpi, Giulia Gallerani, Ivan Vannini, Samanta Salvi, Irene Azzali, Mattia Melloni, Sara Salucci, Michela Battistelli, Patrizia Serra, Roberta Maltoni, William C. Cho, Francesco Fabbri

**Affiliations:** ^1^ Biosciences Laboratory, IRCCS Istituto Romagnolo per Lo Studio Dei Tumori (IRST) “Dino Amadori”, Meldola, Italy; ^2^ Biostatistics and Clinical Trials Unit, IRCCS Istituto Romagnolo per Lo Studio Dei Tumori (IRST) “Dino Amadori”, Meldola, Italy; ^3^ Cellular Signalling Laboratory, Department of Biomedical and NeuroMotor Sciences (DIBINEM), University of Bologna, Bologna, Italy; ^4^ Department of Biomolecular Sciences, University of Urbino Carlo Bo, Urbino, Italy; ^5^ Department of Medical Oncology, IRCCS Istituto Romagnolo per Lo Studio Dei Tumori (IRST) “Dino Amadori”, Meldola, Italy; ^6^ Department of Clinical Oncology, Queen Elizabeth Hospital, Kowloon, Hong Kong, China

**Keywords:** breast cancer, extracellular vesicles, plasma, liquid biopsy, biomarkers

## Abstract

Breast cancer (BC) is the most commonly diagnosed malignant tumor in women worldwide, and the leading cause of cancer death in the female population. The percentage of patients experiencing poor prognosis along with the risk of developing metastasis remains high, also affecting the resistance to current main therapies. Cancer progression and metastatic development are no longer due entirely to their intrinsic characteristics, but also regulated by signals derived from cells of the tumor microenvironment. Extracellular vesicles (EVs) packed with DNA, RNA, and proteins, are the most attractive targets for both diagnostic and therapeutic applications, and represent a decisive challenge as liquid biopsy-based markers. Here we performed a study based on a multiplexed phenotyping flow cytometric approach to characterize BC-derived EVs from BC patients and cell lines, through the detection of multiple antigens. Our data reveal the expression of EVs-related biomarkers derived from BC patient plasma and cell line supernatants, suggesting that EVs could be exploited for characterizing and monitoring disease progression.

## Introduction

Breast cancer (BC) is the most commonly diagnosed malignant tumor in women worldwide, and the leading cause of cancer death in the female population. Although it has been calculated that in Europe, between 2014 and 2019, cancer mortality rate has declined steadily by about 8.7% (Malvezzi et al., 2019), an incidence of 2,261,419 cases and 684,996 deaths were reported in 2020, remaining an alarming concern for public health (Sung et al., 2021). In fact, despite improved clinical management resulting in better prognosis, up to 30% of node-negative BC patients and a larger part of patients with node-positive carcinoma, develop distant metastases after several years from the time of primary tumor detection and surgical resection (Barone et al., 2020). This provides only a relatively poor chance for successful treatments and survival, and identification of new molecular markers for diagnosis and prognosis, especially in a metastatic setting, and for development of innovative therapeutic molecules, are necessary. Furthermore, BC is characterized by a considerable tissue heterogeneity, showing distinct clinical and biological features, which make tumors respond differently to treatments and adverse in their management. In the last years, molecular profiles have been largely explored, providing a well-established classification of BCs into four well-settled subtypes: Luminal A, Luminal B, Basal-like, and human epidermal growth factor receptor 2 (Her2)-enriched. In addition, BC staging also provides useful information about appropriate treatment options, due to its ability to estimate prognosis at each tumor stage. In particular, the Tumor-Node-Metastasis (TNM) system represents an attempt to classify cancer based on the major morphological attributes of malignant tumors that were thought to influence disease prognosis: size of the primary tumor (T), presence and extent of regional lymph node involvement (N), and presence of distant metastases (M) (Singletary and Connolly, 2006; Bandini and Fanini, 2019).

Neovascularization has become a pivotal aspect of tumor and metastasis growth, involving endothelial cell (EC) proliferation, migration, and vascular formation (Chen et al., 2019). In the last years, research has narrowed its attention to the study of the tumor microenvironment (TME) as a target for cancer therapy. In fact, chemoresistance of tumor cells and the development of metastases are no longer due entirely to their intrinsic characteristics, but are also regulated by signals derived from cells of TME. Secreted factors from cancerous cells enable the recruitment of several types of cells required to form the TME, contributing to the formation of a premetastatic niche and to development of chemoresistance (Madden et al., 2020). Tumor stromal cells, including fibroblasts, immunoinflammatory cells, vascular EC and other components of TME, as well as the extracellular matrix, not only play a crucial role in cancer response to therapies, but also orchestrate cancer proliferation, invasion, and metastasis. In particular, ECs are the building pillars of vessels and as such are key players in sprouting angiogenesis (Draoui et al., 2017). Recently, several models and analysis tools have been developed to investigate the crosstalk between mammary cells and neighboring vascular ECs, in order to explore their potential applications in basic research and drugs development. In fact, it could be useful to establish new approaches to develop anti-angiogenic strategies, which represent the few available therapies against the most aggressive BCs (Devadas et al., 2019; Kourti et al., 2020).

Since most of the current methods used for diagnosis and prognosis of cancer are expensive, invasive, and time consuming, new diagnostic panels need to be investigated to make the process less invasive, more cost-effective, and rapid. Among the most promising potential diagnostic targets, extracellular vesicles (EVs) are nanometer-sized, lipid membrane-enclosed vesicles released by many types of cells and classified by different size, components, and functions (Xue et al., 2021). EVs are normally distinguished into three main classes: microvesicles produced through outward budding and fission of the plasma membrane, exosomes derived from endosomes and fusion of multivesicular bodies with the plasma membrane, and apoptotic bodies released as blebs from apoptosis undergoing cells (Wu et al., 2019). Importantly, EVs are the most attractive targets for both therapeutic and diagnostic applications, especially because they are enriched in a large batch of body fluids such as breast milk (Shah et al., 2021), blood plasma (Hu H. et al., 2021), saliva (Hoshino, 2021), urine, serum (Salvi et al., 2021), and cerebrospinal fluid (López-Pérez et al., 2021), becoming an excellent source of potential biomarkers. All cell types are expected to secrete EVs, but their main functions remain to be fully understood. In particular, exosomes are membrane-bound vesicles, 50–200 nm in size, secreted from cells *via* a multivesicular-body endocytic process. This vesicles population has been proposed to perform main functions, among which they are counted to support processes to eliminate DNA, RNA, or protein content that could be detrimental to cell viability, to maintain a cell-to-cell communication system by delivering cargo to a recipient cell, or even to develop a mechanism for surveying cell content for viral infections (Sempere et al., 2017; Jayaseelan, 2020). EVs are enriched in proteins involved in the vesicles’ traﬃcking, cell surface receptors such as tumor susceptibility gene 101 (TSG101), integrins and a number of tetraspanins such as CD9, CD53, CD63, CD81, and CD82 (Burgio et al., 2020). The study of exosomes is relatively difficult and, as referred by the International Society of Extracellular Vesicles (ISEV), the assignment of a specific biogenesis pathway to EVs remains not easy to establish as it could be validated only through a live imaging assay of EV release. Accordingly, due to current technical limitations, almost all studies are unable to isolate and investigate a pure population of exosomes (Romano et al., 2021). Despite being a validated source of biomarkers, liquid biopsy (LB) has not yet succeeded in becoming part of the standard clinical practice in BC patients (Chan et al., 2021). The deepening of isolation and analysis of EVs is essential for understanding their biological roles and for investigating their potential clinical use. Several methods have been developed thus far, but with some limitations (Jong et al., 2017).

The present work aimed at identifying new BC tumor biomarkers through an easy and fast approach, based on a multiplexed phenotyping of EVs released from 30 plasma samples of 10 BC patients. Second, results obtained from patients revealed adhesion molecules markers usually present on the surface of circulating endothelial cells, prompting us to investigate also cell models. Through the analysis of supernatant of BC cell lines, cultured alone or with ECs, we aspired to find clues regarding: 1) EV origin subtypes comparing biomarkers found in plasma and in BC cell cultures and 2) cancer-normal cell interplay, detecting potential marker expression changes in co-culture conditions. EVs were isolated by size exclusion chromatography (SEC) and characterized by a bead-based cytofluorimetric method able to simultaneously detect 37 surface exosomal-related proteins.

## Materials and Methods

### Cell Cultures

The human breast carcinoma cell lines, MDA-MB 453 (Her2-enriched subtype) and MCF-7 (Luminal A molecular subtype) were purchased from ATCC (ATCC; Manassas, Virginia, United States), and cell lines T-47D (Luminal A molecular subtype) and HUVEC (Normal Primary Human Umbilical Vein Endothelial Cells) were purchased from zooprophylactic Institute of Genova (Italy). MDA-MB 453 were maintained in Leibovitz’s L-15 medium (ATCC 30-2008, United States). MCF-7 were maintained in EMEM medium (ATCC 30-2003). T47D were maintained in DMEM High Glucose (Euroclone, Italy). HUVEC were maintained in M199 medium (Sigma Aldrich, Merck, Germany). Each medium was supplemented with FBS exosome-depleted (Gibco, Thermo Fisher Scientific, United States) to a final concentration of 10%, according to the information sheet of the manufacturer. Penicillin-streptomycin (PAA, Carlo Erba Reagents, Italy) to a final concentration of 1% and MycoZap Prophylactic (Lonza Group Ltd., Switzerland) to a final concentration of 0.002% were added to all media. The cultures were maintained in an incubator Heraeus, in an atmosphere composed of 95% air and 5% CO_2_, except for MDA-MB-453 that required a free gas exchange with atmospheric air. Every 4 days we proceeded to the sub-cultivation of cell lines by using Trypsin-EDTA (Life Technologies, United States). Cell lines were tested every 2 months with MycoAlert^™^ *Mycoplasma* Detection Kit (Lonza Group Ltd., Switzerland) to check a possible contamination by *mycoplasma*.

### Patient Sample Collection

The study was conducted on 21 individuals: 10 BC patients diagnosed with early-stage BC enrolled between 2013 and 2014, as well as 11 healthy donors. The samples were enrolled at the Istituto Scientifico Romagnolo per lo Studio e la Cura dei Tumori IRST. Peripheral whole blood was collected at three time points: 1 day before surgery (A), 1 month after surgery (B), and after adjuvant therapy/6 months after surgery (C). None of the patients underwent neoadjuvant therapy or had detectable metastasis at diagnosis. Histological and clinical characteristics are listed in Table 1. Written informed consent was obtained from all subjects before sample analyses. The study was approved by the Ethical Committee of our Institute, Romagna Ethics Committee (CEROM) of Meldola (IRSTB008) and conducted in accordance with the Declaration of Helsinki. Healthy donors were enrolled at the Istituto Scientifico Romagnolo per lo Studio e la Cura dei Tumori IRST and were matched to BC patients for age classes (all female with an average age of 55).

**TABLE 1:** Clinical pathological characteristics of patients. Tumor stage was reported based on the tumor (T), lymph node (N), and metastasis (M) system.

Patient	Age	Subtype	Histology	T	N (positive/asported)	M	Grade	Vascular invasion
1	57	TNBC	Ductal infiltrant	2	0 (1)	0	3	Yes
2	75	LumA	Ductal infiltrant	1b	0 (2)	0	2	No
3	43	TNBC	Ductal infiltrant	1c	1a (1/35)	0	3	No
4	58	TNBC	Ductal infiltrant	2	1a (2/28)	0	3	Yes
5	59	LumA	Ductal infiltrant	1b	0(1)	X	3	Yes
6	43	TNBC	Ductal infiltrant	1c	0 (1)	X	3	No
7	53	LumA	Ductal infiltrant	2	1a (1/15)	X	2	Yes
8	57	LumA	Ductal infiltrant	1b	0 (1)	X	1	No
9	59	LumA	Lobular	1c	0 (2)	0	2	No
10	54	LumA	Ductal infiltrant	1c	0 (1)	X	2	No

### Plasma and Supernatant Collection

Approximately 5 mL of whole blood were collected in EDTA tubes and centrifuged at 1,000 × g for 15 min, followed by a second centrifugation at 1,500 × g 10 min for obtaining plasma. The blood was collected from all individuals before any surgical intervention, 1 month after surgery and after adjuvant therapy/6 months after surgery. Plasma samples were conserved at −80°C until use. Approximately 30 ml of supernatant of BC controls and co-cultured cells were collected 48 h post co-culture and immediately processed to isolate EVs.

### Co-Culture Experiments

Transwell Permeable Supports (Corning, United States) with a 0.45 μm polycarbonate membrane were used in the co-culture model system to separate BC and HUVEC cells into different compartments. HUVEC cells were seeded into a six well plate (lower chamber) and an equivalent number of BC cells (ratio 1:1) were seeded into the transwell insert, which was then placed directly over the 6-well plate containing the HUVECs. BC and HUVEC controls were seeded separately into a six well plate. All cells were maintained in FBS exosome-depleted medium. Two independent experiments were performed and all the 3 cell lines were used (control groups, *n = 3* and co-cultured groups, *n* = 3). Cells were incubated for 48 h and then washed in PBS 1X and harvested for further analysis.

### Isolation of EVs From Cell Culture Medium and Plasma of BC Patients

30–40 mL of supernatants from BC and HUVEC cells containing exosome-depleted FBS (Gibco, Thermo Fisher Scientific, United States) were collected after 48 h co-culture, centrifuged at 300 × g for 10 min, filtered by 0.22 µm syringe to exclude cell debris and further purified by centrifugation for 15 min at 1,000 × *g* and for 15 min at 2,000 × *g*. Subsequently supernatants were concentrated through Centricon Plus-70 centrifugal filter devices (Merck Millipore, Darmstadt, Germany). For BC patients, 500 µL of plasma were used. For EVs isolation, qEV10 Size Exclusion Columns (70 nm, Izon Science) were used. After rinsing the columns with PBS 1X, 300–500 µL of concentrated culture medium were applied on the top of a qEV column and 0.5 mL fractions were collected. Four vesicles-enriched fractions (7–10) were firstly analyzed, then EVs content analysis was performed on fraction 8 after nanoparticle tracking analysis (NTA) evaluation.

### Nanoparticle Tracking Analysis

NTA was used to determine particles size and estimate number/ml of isolated EVs from subjects and cell lines. EVs were characterized by NTA with a NanoSight NS300 (Malvern Instruments, United Kingdom), equipped with NTA 2.3 analytical software laser. Five 30 s videos were recorded per sample in light scatter mode with a camera level of 14 and from these the software calculated the mean and the mode diameter (nm) and EV concentrations. Software settings for analysis were kept constant for all measurements. All samples were diluted in 0.1 µm filtered PBS to an appropriate concentration before analysis. Based on the data obtained at NTA, which highlighted the fraction eight to be more concentrated and homogenous, we proceeded with downstream analyses with fraction eight for all the samples. Data were analyzed with the NTA version 2.3.

### Extraction of Proteins

Proteins were concentrated from the fraction eight of EVs obtained from plasma of patients and from supernatant of cell lines. Then total proteins were extracted, keeping samples on ice, with 1X RIPA lysis buffer (Santa Cruz Biotechnology, United States) with the addition of 10 µL of PMSF, 10 µL of sodium orthovanadate and 15 µL of protease inhibitors per ml of 1X RIPA lysis buffer, as recommended by the manufacturer’s protocol. The lysates were centrifuged at 4°C at 13,000 × g for 30 min. Then, the supernatant was transferred to another tube. Proteins were subsequently quantified following the protocol of the BCA Protein Assay (Pierce, Thermo Fisher Scientific, United States) and using a Multiscan EX microplate reader (Thermo Fisher Scientific, United States), with a wavelength filter of 490 nm.

### Protein Expression Analyses

Western blotting was used to evaluate the expression of the exosome markers CD9, Alix, CD81, TSG-101, and Calnexin. Twenty µg of proteins were denatured and separated by electrophoresis using Criterion TGX Stain Free Gel Precast 4–20% (Bio-Rad Laboratories, CA, United States) and Laemmli Sample Buffer (Bio-Rad Laboratories, CA, United States) with 5% of *β*-mercaptoethanol (Carlo Erba Reagents, Italy), in 1:1 ratio with the sample. The electrophoretic run was performed at a constant voltage of 180 V in a TRIS/*Glycine*/SDS 1X buffer (Bio-Rad Laboratories, CA, United States). Proteins were then transferred onto a PVDF membrane (Trans-Blot Transfer Turbo midi-format 0.2 µm; Bio-Rad Laboratories) using the Trans Blot Turbo System (Bio-Rad Laboratories, CA, United States). The membrane was subsequently incubated for at least 2 h at room temperature in a solution of Tween 20 (Bio-Rad Laboratories, CA, United States) at 0.1% and 1X Dulbecco’s Phosphate Buffered Saline (Invitrogen, Thermo Fisher Scientific, United States) supplemented with 5% milk powder (Blotting Grade Blocker Non-Fat Dry Milk; Bio-Rad) in order to facilitate the saturation of non-specific binding sites. Primary antibodies and dilutions used are the following: CD9 (D8O1A, Cell Signaling, United States) 1:1,000, Alix (3A9, Cell Signaling, United States) 1:1,000, CD81 (D4, Santa Cruz, United States) 1:1,000, TSG-101 (T5701, Sigma-Aldrich, Merck, Germany), 1:1,000 and Calnexin (2,433, Cell Signaling, United States) 1:1,000. Secondary antibodies and dilutions used are the following: Goat anti-rabbit IgG-HRP and Goat anti-mouse IgG-HRP (Santa Cruz, United States) 1:5,000, Precision Plus Protein Western C StrepTactin-HRP Conjugate (Bio-Rad Laboratories, CA, United States) 1:10,000. Blocking and immunological reactions were performed in accordance with the protocol Western Immunoblotting of Cell Signaling. Images were developed through the SuperSignal West Femto (Pierce, Thermo Fisher Scientific, United States) or Clarity West-ern ECL Substrate (Bio-Rad Laboratories, CA, United States) and acquired through Chemidoc (Bio-Rad Laboratories, CA, United States).

### Bead-Based Multiplex Exosome Flow Cytometry Assay

Samples were subjected to bead-based multiplex EV analysis by flow cytometry (MACSPlex Exosome Kit, Miltenyi Biotec, Bergisch-Gladbach, Germany). The MACSPlex Exosome Kit (Miltenyi Biotec, Germany) allows the detection of 37 exosomal surface epitopes (CD3, CD4, CD19, CD8, HLA-DR, CD56, CD105, CD2, CD1c, CD25, CD49e, ROR1, CD209, CD9, SSEA4, HLA-BC, CD63, CD40, CD62 P, CD11c, CD81, MCSP1, CD146, CD41b, CD42a, CD24, CD86, CD44, CD326, CD133/1, CD29, CD69, CD142, CD45, CD31, CD20, and CD14) plus two isotype controls (REA and IgG1). The MACSPlex Exosome Detection Reagents for CD9, CD81, and CD63 were used to label the captured EVs. EV-containing samples were processed as follows: vesicles were diluted with MACSPlex buffer (MPB) to a final volume of 120 μL, then 15 µL of MACSPlex Exosome Capture Beads (containing 39 different antibody-coated bead subsets) were added to each sample. One negative/blank control (MACSPlex Buffer only) was used in each run experiment to determine non-specific signals. For counterstaining of particles bound by capture beads with detection antibodies, 5 µL of each APC-conjugated anti-CD9, anti-CD63, and anti-CD81 detection antibody were added to each sample, then they were incubated on an orbital shaker at 450 rpm protected from light for 1 h at room temperature. Next, samples were washed with MPB and incubated on an orbital shaker at 450 rpm protected from light for 15 min. Subsequently, a further MPB washing was performed and flow cytometric analysis was carried out through a BD FACSCanto equipped with two lasers, 488 nm and 630 nm (Becton Dickinson, San Diego, CA, USA), recording a minimum of 50 events for each population of specific beads. The detection of FITC, PE, and APC fluorophores were measured for each sample. For each sample, the 39 bead populations (37 exosomal surface epitopes + 2 isotype controls) were distinguished by different fluorescence intensities detected in the FITC, PE, and APC channels. Final analysis was performed through the corresponding software (BD FACSDiva): from the raw median fluorescence intensity (MFI) of each marker was subtracted the MFI of the negative control used in the same run experiment.

### Transmission Electron Microscopy

EVs isolated from the cell lines supernatants were adsorbed to form VAR carbon coated 200 mesh grids (Agar Scientific Ltd., Stansted, United Kingdom) for 2 min, and briefly rinsed in filtered PBS 1X. Vesicles on grids were immediately fixed with 2.5% glutaraldehyde for 1 min and then negatively stained with 2% (wt/vol) Na-phosphotungstate for 1 min. The observations were carried out by means of a Philips CM10 transmission electron microscope at 80 kV.

### Data Analysis

The images of the Western blot were acquired through Chemidoc (Bio-Rad Laboratories) and BD FACSDiva software was used to perform Flow Cytometry analysis.

### Statistical Analysis

The aim of the study was to evaluate the technical feasibility of a new workflow to investigate the potential role of EVs in early diagnosis of BC. Descriptive statistics were reported as proportions and median values (range). Non-parametric ranking test (Median test) was used to compare continuous data. MACSPlex results were analyzed by *t* test or analysis of variance (ANOVA) for repeated measures. The associations between continuous variables were determined using Spearman correlation analysis. To generate heatmaps of data, data were exported to comma separated files, which were subsequently imported into R Software for further analysis and data visualization. Mann Whitney *U* test (non-parametric ranking test) was used to compare EVs mean diameter and EVs mode diameter between healthy volunteers (*n* = 11) and patients (*n* = 10). Robust rank-based ANOVA (ATS) was used to detect a time effect on EVs mean diameter and EVs mode diameter in patients, and multiple comparisons (again ATS) were performed to determine which time points differed. Due to the explorative nature of the study, no formal sample size calculations were performed and no multiple test corrections were made. All *p* values were based on two-sided testing, and *p*-values < 0.05 were considered statistically significant. Statistical analysis was carried out using SAS software, version 9.4 (SAS Institute, Cary, NC, United States) and R statistical package version v 4.0.0 (R Foundation for Statistical Computing, Vienna, Austria) with nparLD package version 2.1.

## Results

### Characterization of EVs Isolated From Plasma

**FIGURE 1:**
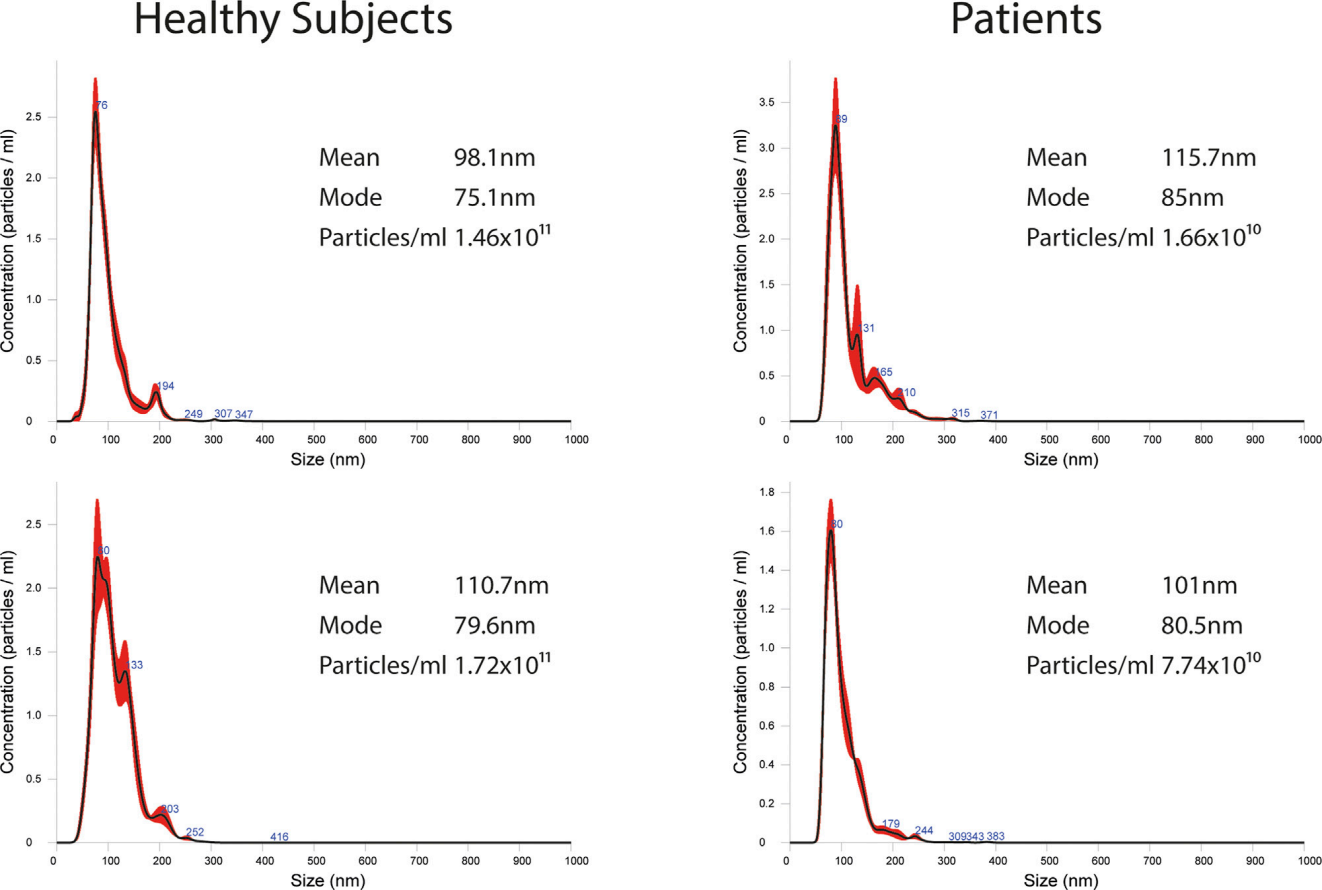
Representative images of NTA profiles analysis of EVs derived from BC plasma samples and healthy donors and corresponding concentration by Nanosight instrument.

**FIGURE 2:**
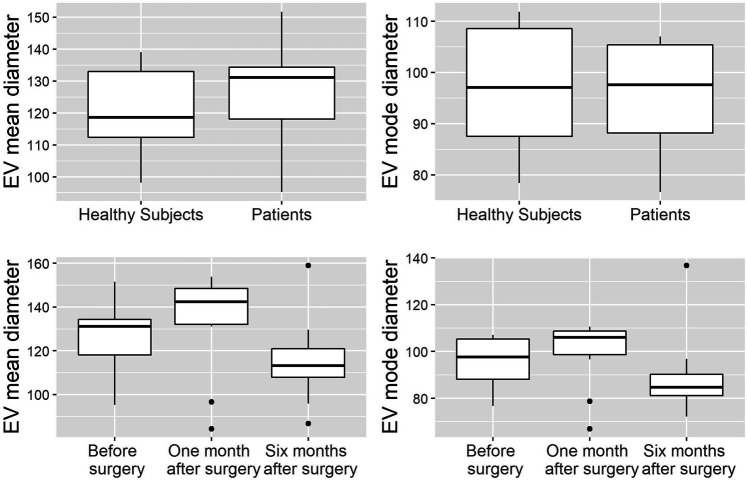
Analysis of EV mean and mode diameters, comparison of healthy subjects vs. patients across three time points. Mann Whitney *U* test was used to compare EVs mean diameter and EVs mode diameter between healthy volunteers (*n* = 11) and patients (*n* = 10). Robust rank-based ANOVA (ATS) was used to detect a time effect on EVs mean diameter and EVs mode diameter.

**FIGURE 3:**
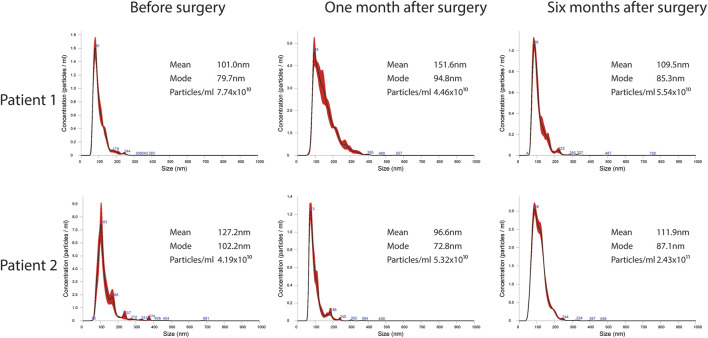
Representative images of NTA profiles analysis of EVs derived from BC plasma samples analyzed at three different time points: pre-surgery, 1 month post-surgery and after adjuvant therapy/6 months post-surgery, and corresponding concentration by Nanosight Instrument.

**FIGURE 4:**
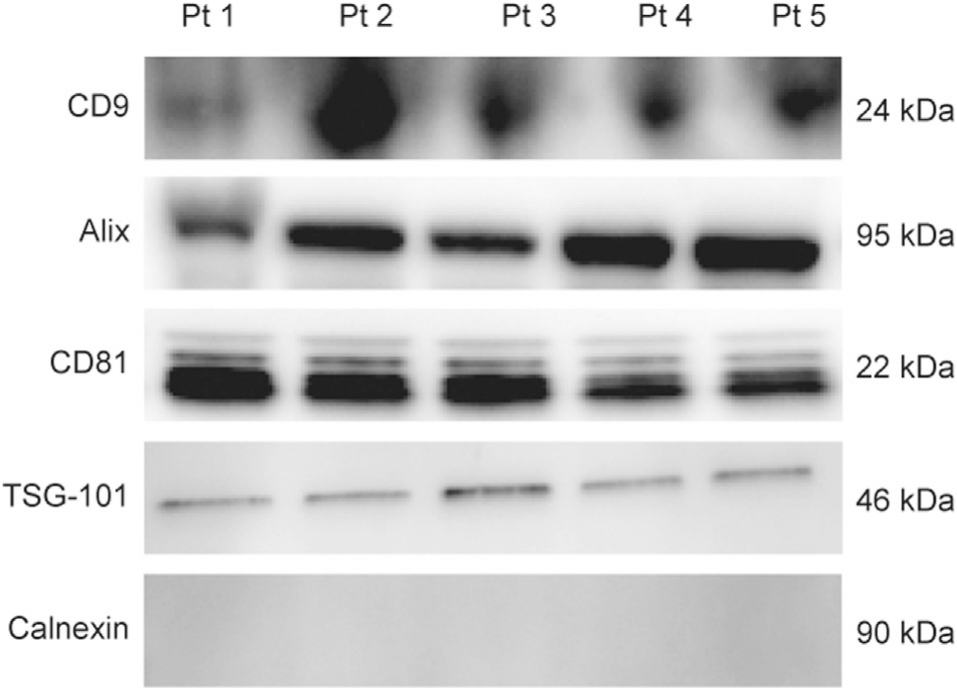
Representative WB analysis of SEC-EVs (fraction 8) derived from plasma of BC patients using EV markers CD9, Alix, CD81, TSG-101, and Calnexin.

**FIGURE 5:**
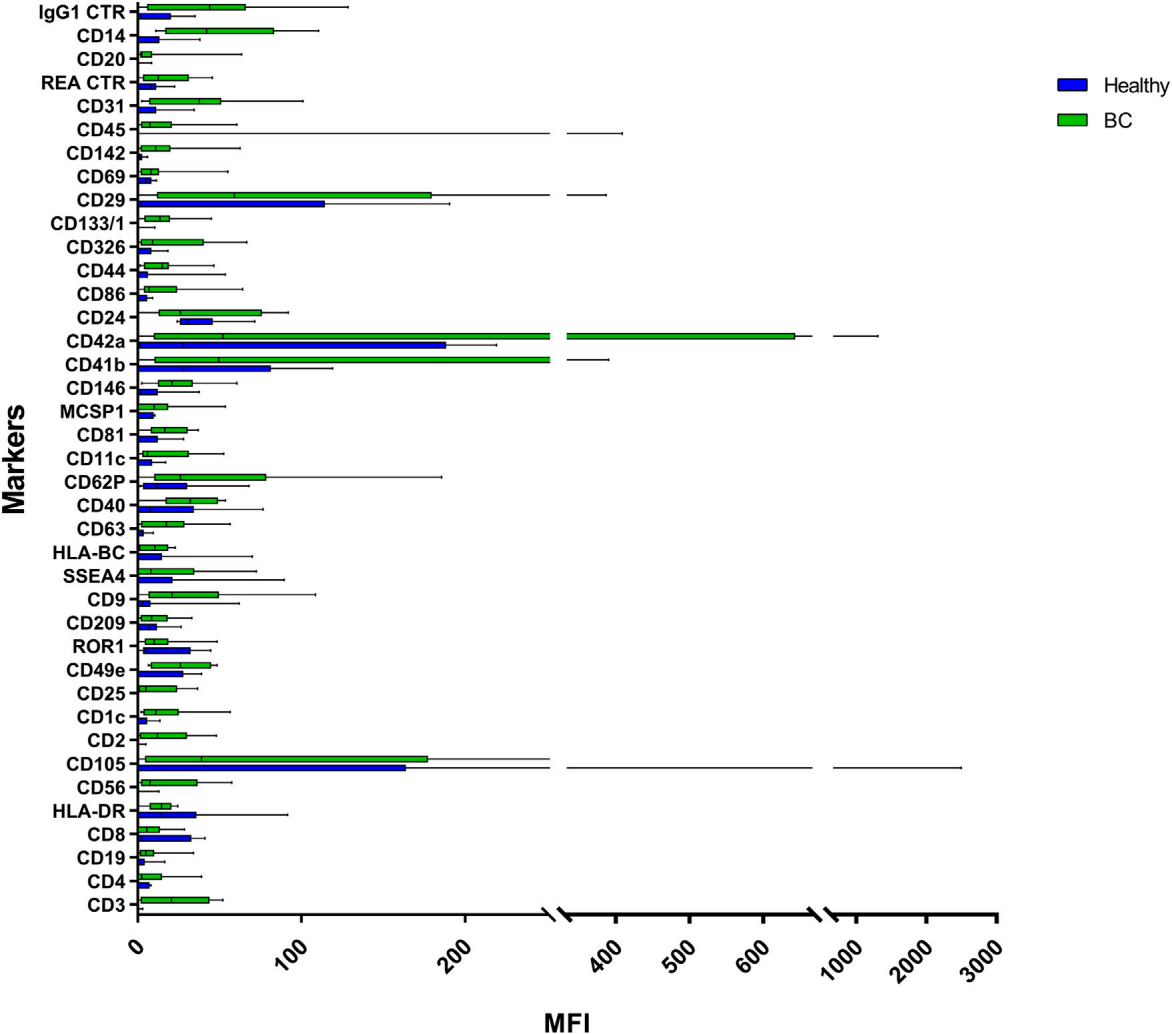
Range from min to max of the Mean Fluorescence Intensity (MFI) for each plasma EVs marker. Plasma from healthy donors in blue; plasma from BC patients in green; values have been normalized to blank control. MFI, mean fluorescence intensity.

**FIGURE 6:**
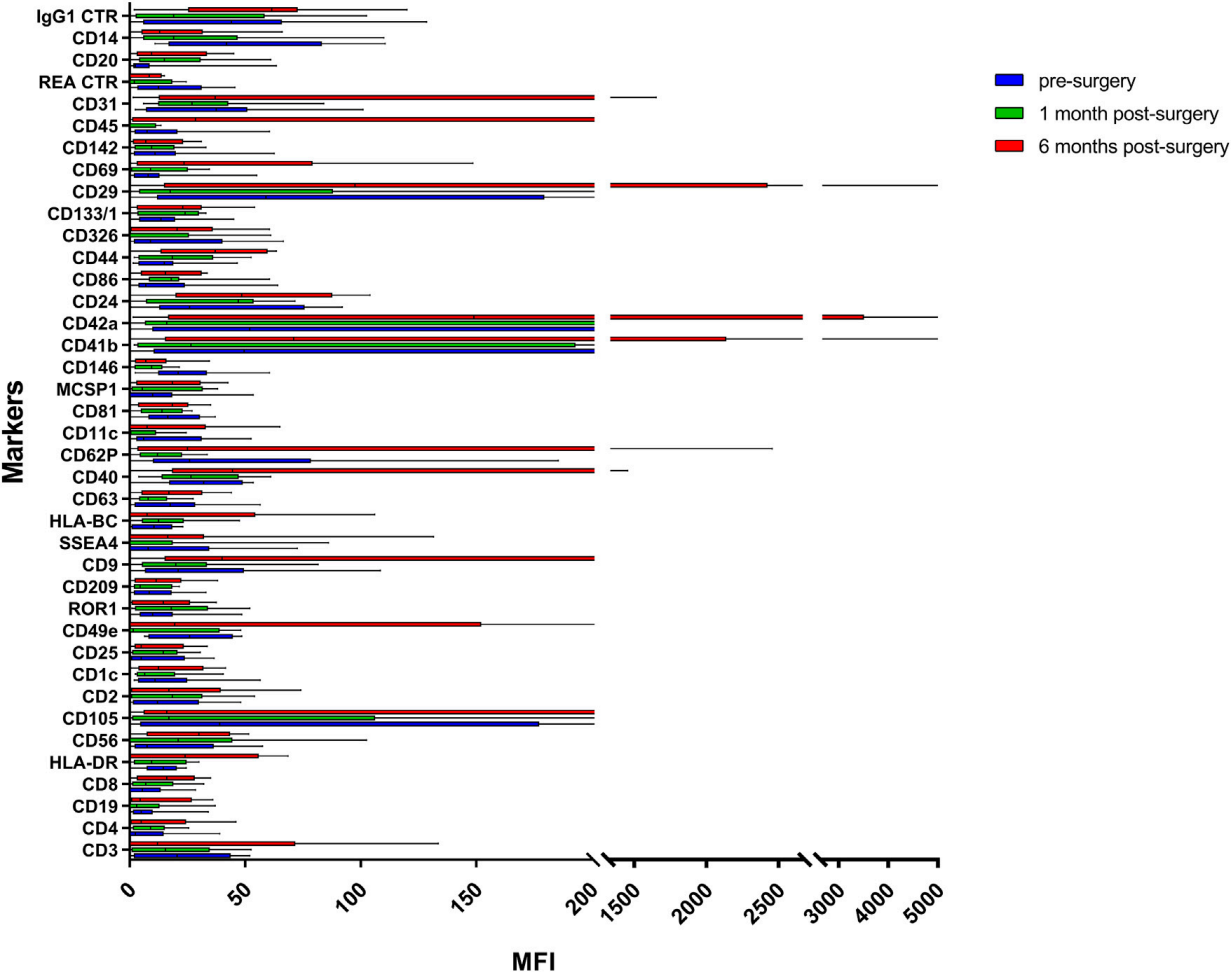
Range from min to max of the MFI for each plasma EVs marker. Plasma from BC patients before surgery in blue; plasma from BC patients 1 month post-surgery in green; plasma from BC patients 6 months post-surgery in red; values have been normalized to blank control. MFI, mean fluorescence intensity.

EVs were successfully isolated from plasma of BC patients and healthy donors through SEC, recently established a reliable EVs-isolation method that allows separation of EVs from a considerable portion of lipoproteins and other plasma components (Brahmer et al., 2019). NTA showed that the EVs from the plasma of BC patients had a mean size of 131.1 nm, range 95.3–151.6 nm, and a mode of 97.6. Concentrations ranged between 4.9 × 10^9^ – 5.43 × 10^10^ particles/ml as shown in Figure 1. EVs from plasma of healthy donors were characterized by a mean size of 118.6 nm, range 98.2–139 nm, and a mode of 97.1. No statistical difference was observed between the EV mean diameters (*p* = 0.403) and the EV mode diameters (*p* = 0.802) of healthy subjects vs. patients (Figure 2). Concentrations ranged between 3.10 × 10^9^ – 1.72 × 10^11^ particles/ml. In vesicles derived from BC patients among three time points (Figure 3), a significant time effect was detected on EV mean diameters (*p* = 0.016), in particular for pre-surgery vs. post-surgery (median EV mean diameter 131.1 nm vs. 142.4 nm, *p* = 0.021) and for 1 month post-surgery vs. 6 months post-surgery (median EV mean diameter 142.4 nm vs. 113.2 nm, *p* = 0.02). No significant time effect was found on EV mode diameter (*p* = 0.052) (Figure 2). The isolated vesicles were analyzed for the presence of exosomal markers confirmed by western blot, showing positivity at different levels for CD9, Alix, CD81, and TSG-101 while they were negative for the expression of Calnexin (Figure 4). Furthermore, they were analyzed through flow cytometry (Figures 5, 6), revealing a variation in the expression of EVs markers between patients and healthy donors and among three different time points of BC patients.

### EV Markers Differentially Expressed in Plasma of BC Patients

Plasma EVs analysis showed that 11 significant markers were able to significantly discriminate between healthy subjects and patients: CD3, CD56, CD2, CD25, CD9, CD44, CD326, CD133/1, CD142, CD45, and CD14 (Table 2). All markers significantly distinguish healthy subjects and BC cases: CD3, CD25, CD56 (*p* < 0.001); CD2, CD9, CD142, and CD14 (*p* < 0.01); CD44, CD326, CD133/1, and CD45 (*p* < 0.05). Statistical results confirmed the trend of tumor samples to have, on average, higher marker values than healthy ones, except for CD45 that decreases its fluorescent intensity in BC cases. Statistical differences were further observed within different time points of BC patients for CD146 (*p* = 0.034) and CD45 (*p* = 0.047) (Table 3). More specifically, both markers were found downregulated 1 month after surgery compared to the first access (CD146 *p* = 0.042 and CD45 *p* = 0.040). Data were further evaluated by heatmap analyses, showing however only a weak clustering of CD42a and CD41b that seemed independent from subtype and time points (Supplementary Figure S1). The expression of EV markers CD105, CD1C, CD62p, CD41b, CD42a, CD326, and CD29 in BC patients was associated with age of patients (Table 4). Among them, CD1c decreased with age while the other antigens increased. In healthy subjects, only CD209 resulted inversely correlated to age, and it decreased along with the increasing of age (*p* = 0.026).

**TABLE 2:** Summary of significant values calculated through analysis of mean values of plasma EVs derived from healthy subjects and BC patients. MFI, mean fluorescence intensity. Unpaired Student’s *t* test was used to perform analysis.

Markers	Healthy mean MFI	BCs mean MFI	*p*-value
CD3	0.273	22.444	0.001
CD56	1.182	17.944	0.001
CD2	0.500	16.333	0.006
CD25	0.000	11.444	0.0009
CD9	8.455	33.444	0.006
CD44	9.591	15.056	0.044
CD326	3.864	21.444	0.044
CD133/1	1.045	14.278	0.019
CD142	1.727	15.000	0.006
CD45	40.318	14.167	0.019
CD14	8.773	49.778	0.006

**TABLE 3:** Summary of significant values calculated through analysis of mean values of plasma EVs derived from BC patients at different time points (before surgical intervention, 1 month after surgical intervention and 6 months after surgical intervention). MFI, mean fluorescence intensity. ANOVA test for repeated measures was used to perform analysis.

Markers	Mean MFI time A	Mean MFI time B	Mean MFI time C	*p*-value
CD146	24.444	8.722	10.111	0.034
CD45	14.167	4.889	104.056	0.047

**TABLE 4:** Association between baseline markers values and age in BC patients. Spearman correlation was used to perform analysis. If r_s_ is positive, age and marker expression are directly/positively proportional; if r_s_ is negative, age, and marker expression are negatively correlated.

	Age	
	r_s_	*p-*value
CD105	0.79	0.006
CD1c	−0.82	0.004
CD62p	0.69	0.029
CD41b	0.72	0.019
CD42a	0.68	0.031
CD326	0.75	0.012
CD29	0.68	0.031

### Characterization of EVs Isolated From BC Cell Lines

**FIGURE 7:**
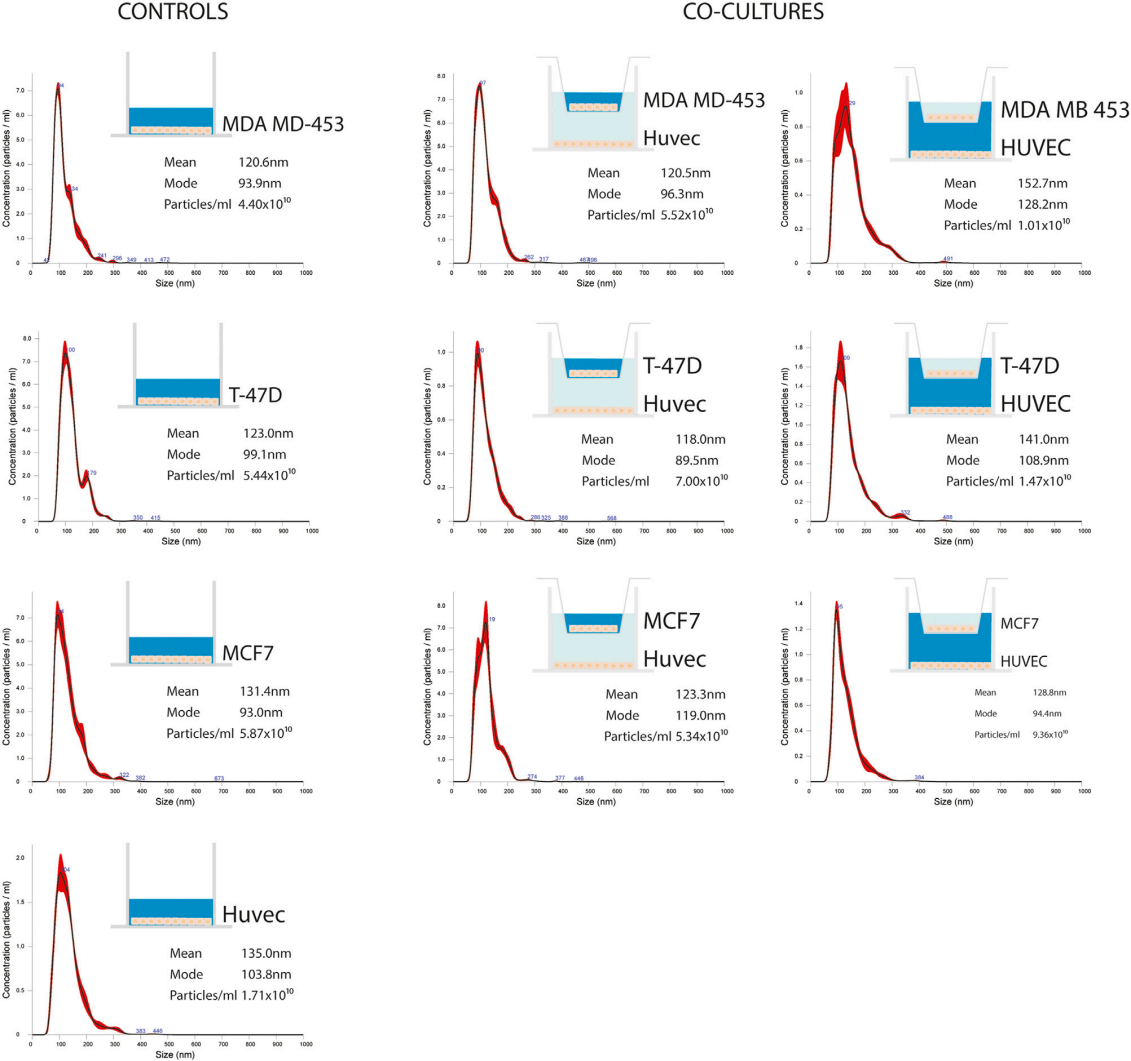
Representative distribution plots of EVs isolated from supernatant of BC cell lines (MDA-MB 453, T-47D, and MCF-7) alone and in co-cultured conditions with endothelial cells HUVEC; each dot plot refers to the corresponding medium analyzed and from which EVs were isolated (in blue). For each plot, there is a corresponding concentration made by Nanosight instrument.

**FIGURE 8:**
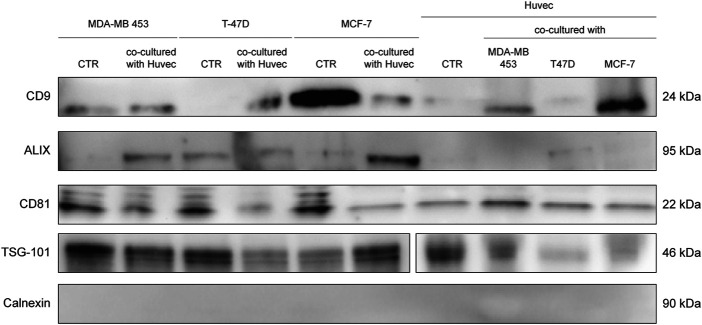
WB analysis of isolated EVs from SEC fraction eight derived from supernatant of BC cells and HUVEC, using EV markers CD9, Alix, CD81, TSG-101, and Calnexin.

**FIGURE 9:**
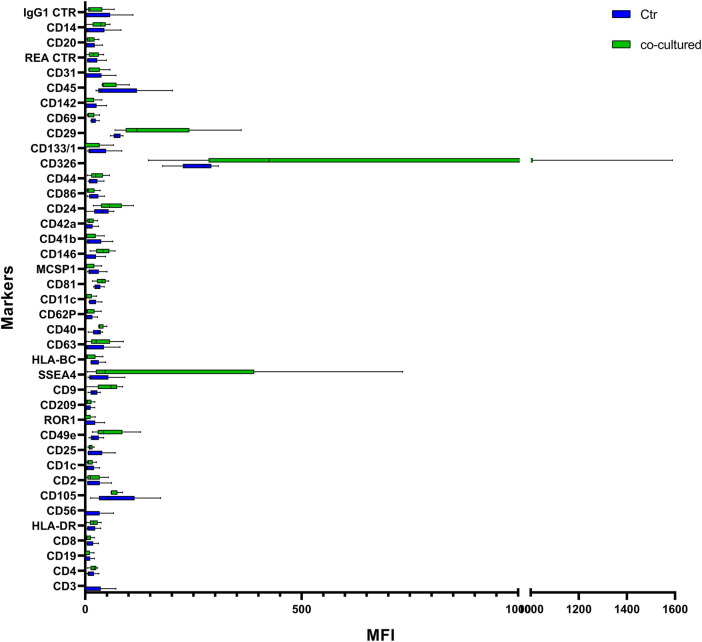
Range from min to max of MFI for each cell EVs marker. Supernatant from cell controls in blue; supernatant from cell co-cultured with HUVEC in green; values have been normalized to blank control. MFI, mean fluorescence intensity.

**FIGURE 10:**
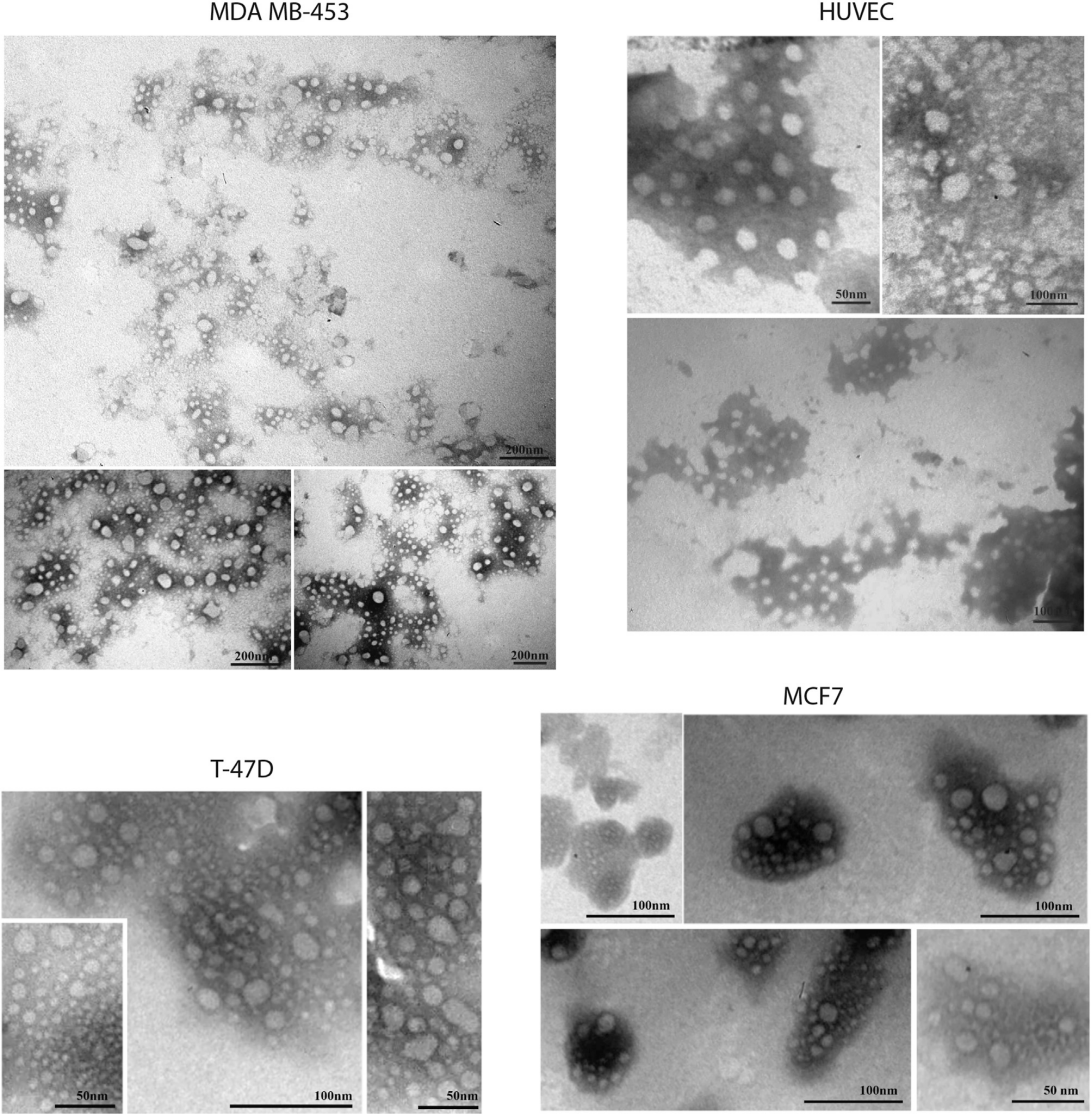
Transmission electron microscopy representative images of SEC-EVs isolated from BC cells and endothelial cells. TEM observations showed numerous EVs between 5 and 100 nm. Scale bar refers to 50–100 nm.

EVs were successfully isolated from BC and HUVEC cell lines through SEC. NTA showed a similar distribution for EVs from three different BC cells: MDA-MB 453 EVs had a mean size of 120.5 nm in controls and 120.6 in co-cultured samples; T-47D EVs had a mean size of 123.1 in controls and 118 in co-cultured samples; MCF-7 EVs had a mean size of 131.4 in controls and 123.3 in co-cultured samples. All cell lines showed concentrations between 4.4 × 10^10^ – 7 × 10^10^ particles/ml as shown in Figure 7. Interestingly, we observed a slight trend for vesicles to increase their production or release in co-cultured cells compared to the controls, in particular for MDA-MB 453 (4.4 × 10^10^ control cells vs. 5.52 × 10^10^ co-cultured cells) and T-47D (5.5 × 10^10^ control cells vs. 7 × 10^10^ co-cultured cells) cell lines. EVs from HUVEC cells were slightly larger compared to those from BC cells, being characterized by a mean size of 134.9 nm in control cells, and by means varying from 118–131.4 nm when co-cultured with BC cell lines, together with lower concentrations between 9.36 × 10^9^ – 1.7 × 10^10^ particles/ml. No significant differences were observed between control and co-cultured cells for vesicle size. Western blot analysis showed that EVs of BC cells and HUVEC were positive, at different levels, for CD9, Alix, CD81, and TSG-101, and negative for the expression of Calnexin (Figure 8). Furthermore, vesicles were analyzed both by flow cytometry and TEM. Flow cytometry analysis revealed a variation in the expression of exosomal markers between co-cultured cells and controls (Figure 9). The morphology of EVs isolated from BC cells was examined through TEM analysis. MDA-MB 453 were characterized by vesicles with sizes between 20–100 nm and not all of them characterized by well-preserved membranes; T-47D cells were defined by preserved membranes and small vesicles of size between 5–30 nm, some with irregular shape. MCF-7 cells were characterized by preserved membranes and EVs with sizes between 5–25 nm. HUVEC showed less conserved membranes and their vesicles were characterized by a size between 20–60 nm (Figure 10).

### EVs Differentially Expressed in Vesicles Derived From BC Cells After Co-culture With Endothelial Cells

Cytofluorimetric EVs analysis showed six significant antigens discriminating between BC cells co-cultured with ECs and BC cells alone with a *p*-value < 0.05: CD4, CD105, CD40, CD146, CD44, and CD29 (Table 5). CD105 (*p* = 0.001) and CD40 (*p* = 0.006) were further confirmed as factors that clearly distinguished EVs derived from BC cells co-cultured with ECs, along with CD81 as correlated to co-cultured phenotype (*p* = 0.0005).

**TABLE 5:** Summary of significant values calculated through analysis of mean values of EVs derived from supernatant of BC cell lines and controls. MFI: mean fluorescent intensity; Ctr: control. Two independent experiments were performed and 3 cell lines were used (control groups, *n* = 3 and co-coltured groups, *n* = 3). Unpaired Student’s *t* test was used to perform the analysis.

Markers	Mean MFI Ctr	Mean MFI co-cultured	*p*-value
CD4	14.833	18.500	0.025
CD105	79.667	69.000	0.025
CD40	26.00	38.000	0.025
CD146	16.333	40.667	0.025
CD44	20.833	28.500	0.025
CD29	73.500	183.000	0.025

## Discussion

In BC, clinicopathological characteristics such as age, grade, stage, and molecular subtypes correlate to different incidences, survival, prognosis, and biology, influencing clinical decisions. In addition to tumor cell biology, an inflammatory microenvironment can be responsible for cancer growth. In fact, to date, it is well known that TME can affect carcinogenesis at various steps, from initiation to progression (Tekpli et al., 2019). Many risk factors have been recognized for BC development including age, family history, genetic mutations, chronic inflammation, obesity, and personal habits. Indeed, cancer development is a complex and progressive process that involves modifications not only in the tumor initiating cells, but also in the surrounding environment constituted by several types of cells and secreted biomolecules (Deshmukh et al., 2019), among which EVs are the key of this interplay.

Equally importantly, the interaction between tumor cells and TME at metastatic sites has been recognized as a key regulator of tumor progression, and a better understanding of the mechanisms through which BC-derived EVs guide secondary metastasis is so crucial (Kim et al., 2020).

TME includes a variety of cell types: fibroblasts, ECs, immune cells, pericytes, adipocytes, and local and bone marrow-derived cells, surrounded by matrix components. Moreover, blood supply plays a pivotal role in cancer progression, allowing access to oxygen and nutrients that support tumor spread and eliminate metabolic waste. In this context, angiogenesis, the process by which new blood vessels arise from pre-existing ones, represents a central step in the progression of tumor growth and metastases dissemination, and the blockade of angiogenesis is a promising challenge for new cancer therapies. Hence, although somewhat partial, studying the possible interaction between ECs and BC cells through *in vitro* studies of EVs could be a fair starting point to solve and more deeply understand the intricate interactions between cancer cells and TME (Bovy et al., 2015). In the future, it should be possible to translate the findings into the clinic after identification of tumor-specific actionable targets and validation of new EV-based markers of prognosis and/or resistance to therapy (Möller and Lobb, 2020).

The release of EVs into the extracellular space means a chance to examine them in body fluids such as blood, urine, liquor, and malignant effusions, making them potential biomarkers for the clinical management of cancer with some notable advantages (Mathew et al., 2020). First of all it is a non-invasive way to recover samples from a number of biologic materials. Secondarily, circulating EVs analysis could represent a “liquid biopsy” with the convenience of not requesting cancerous tissue or the partial or total removal of a tumor to access its molecular information, and the capability to monitor cancer progression due to consecutive repeatable sampling (Lucchetti et al., 2019). However, one of the main issues highlighted by the scientific community concerns the absence of a standardized protocol for enrichment and characterization of EVs (Lane et al., 2018), although numerous methods have been developed in order to investigate their behavior. Generally, EVs can be differentiated by size, density, and protein composition, but it is still demanding to easily fractionate EVs and microvesicles due to the marked similarity of their composition. EVs can be isolated through a variety of techniques, such as centrifugation (high speed, differential, and density-gradient), membrane affinity columns, SEC, filtration, and precipitation. Many of these methods are characterized by poor purity and consistence (Hu T. et al., 2021). Besides the most commonly used approaches to obtain EVs, which have some limitations (Greening et al., 2015; Lobb et al., 2015), several other strategies, including flow cytometry ones, are gaining interest (Maia et al., 2020; Marchisio et al., 2020). Hence, before translating into clinics, methods such as that herein reported (Salvi et al., 2021) need to be tested, and clinical validations need to be performed. In order to investigate blood-related TME and discover new potential disease-related biomarkers through a recent approach, we performed a small case study, highlighting the feasibility of the detection and characterization of BC-derived EVs. We used both plasma samples of BC patients and BC cell lines co-cultured with ECs. Patient samples were taken before surgery, and after 1 and 6 months after surgery, together with adjuvant therapy, to investigate the value of EVs as cancer markers in this clinical setting during the earliest stage of the disease, in order to discover possible biomarkers to monitor patients in the first months after surgery and/or during therapy. We first isolated EVs through SEC, and then characterized EVs through NTA, WB, and a multiplexed phenotyping cytofluorimetric approach able to detect 37 exosomes-related antigens. NTA analysis of EVs derived from BC patients did not show a variation in the dimensions of vesicles compared to that of healthy subjects, but significant results were observed among the three different time points, especially in vesicles analyzed 1 month post-surgery. The MACSPlex-based characterization showed that CD3, CD56, CD2, CD25, CD9, CD44, CD326, CD133/1, CD142, CD45, and CD14 markers were differentially expressed, with significance, between healthy subjects and patients. Interestingly, CD146 and CD45 were found significantly deregulated at the three different time points. In particular, both CD146 and CD45 expression levels were reduced in EVs 1 month after surgical resection, suggesting that these markers could have a value for monitoring disease after surgical resection, therapy, and progression. In line with these results, CD146 is considered a hallmark of tumor progression and metastasis, especially in TNBC (Li et al., 2021) and CD45 was found deregulated in breast stroma of BC patients at early stages also (Marino et al., 2020). Furthermore, CD146 is a well-known endothelial cell lineage marker. Firstly identified as a melanoma cell adhesion molecule (MCAM), it is overexpressed in many tumors and implicated in vascular and lymphatic metastasis (Wang et al., 2020). CD146 is a molecule known to modulate cell-cell adhesion and to bind to extracellular matrix proteins or other transmembrane proteins, such as VEGFR2, and the secretion of CD146-enriched EVs was reported to be associated with metastatic process, mediating their interaction with specific ligands on endothelial cells of metastatic organs (Ghoroghi et al., 2021). Since we observed a CD146 decrease during the time in which patients did not progress in 5 years, it is tempting to hypothesize that a reduction of this marker could be related to a better prognosis. We further reported deregulated markers whose presence suggests a peculiar asset of EVs in aging cancer patients. Specific age-related EV markers is a field that would be worth being studied, in particular to more deeply understand the well-known association between cancer and aging.

In order to shed some light on the EV epitopes we found in the clinical setting, we set up an experimental plan *in vitro* that could confirm the results obtained for deregulated markers observed. In our cell models, the interaction of BC cells with ECs firstly seems to lead to a slight increase of EV release, compared to the number of EVs produced by cancer or HUVEC cells. A range of markers were identified with increased signal intensity in samples co-cultured with ECs: CD4, CD105, CD40, CD146, CD44, CD29, and CD81, with only CD44 and CD146 variation found common in both patients and cell models. Although results between BC cells and patients were not completely comparable, most probably due to the different nature of samples, the increase of these markers in BC cells-released vesicles may hint some interesting thoughts. The interaction of cancer cells with a simplified normal microenvironment (herein streamlined by HUVEC cells) may trigger the production of EVs exhibiting antigens related to endothelial/neo-angiogenetic and/or aggressiveness features. Indeed, CD146 and CD44 have been related to neo-angiogenesis, cell proliferation, cell survival, cytoskeletal changes, and cellular motility (Chen et al., 2018). This may suggest an involvement of ECs in the acquisition of a more aggressive behavior of cancer cells, as already reported (Hwang et al., 2020). Despite not being significantly different in BC patients vs healthy donors, CD146 expression decreased in our BC patients over time, as previously reported here, suggesting a role in tumor related neo-angiogenetic processes. On the other hand, CD44 was altered both in patients vs healthy subjects and in cell models, suggesting that this could be a cancer-related marker of spreading and prompting a deeper investigation of this antigen in BC patient EVs. Furthermore, a recent study showed that CD44 circulating tumor endothelial cells were associated with poor prognosis in pancreatic ductal adenocarcinoma after radical surgery (Xing et al., 2021). Indeed, the identification of circulating factors that might be responsible for influencing and spreading vessels formation, modulating angiogenesis and aggressiveness, it is of great clinical interest. It is increasingly necessary to support the study of new biomarkers of angiogenesis and metastatic spread, with the final aim of an accurate disease monitoring targeted towards personalized medicine. In agreement, a study reported a high percentage of patients exhibiting high tumor-infiltrating lymphocytes with CD3 positivity exhibited pathological response to neoadjuvant chemotherapy (Gomez-Μacias et al., 2020). From this point of view, in the future, the herein feasible detection of CD3-positive EVs, potentially secreted by CD-positive infiltrating lymphocytes, could also be investigated and utilized as a marker of response. In summary, we performed a small preliminary and feasibility study to investigate useful biomarkers possibly exploitable for diagnostic and monitoring intent, as already reported for monitoring therapies in various types of cancer (Stevic et al., 2020). Moreover, although dissimilar from the clinical setting in terms of expression of some markers, the results from *in vitro* analysis of EVs suggested the implication of CD44 and CD146 in biological processes involved in breast tumor and microenvironment interplay. The fact that we observed significant results already in a small but well monitored series of patients recommends continuing in this direction. However, since we are aware of the limitations of this study, principally due to the small size of our patient cohort, future validation studies with a larger set of patients are clearly needed.

## Data Availability

The raw data supporting the conclusions of this article will be made available by the authors, without undue reservation.
