# Supplementary figures and images for: Early Detection and Investigation of Extracellular Vesicles Biomarkers in Breast Cancer
Source: Front Mol Biosci. 2021 Nov 8;8:732900. doi: 10.3389/fmolb.2021.732900 (PMC8606536; doi:10.3389/fmolb.2021.732900)

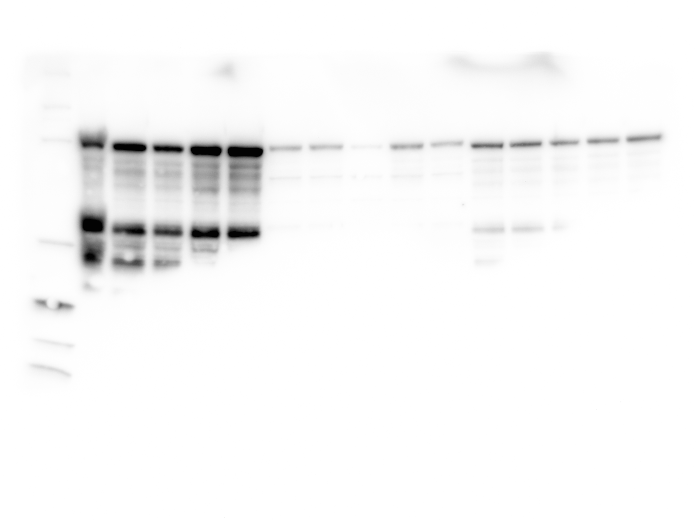

Supplement: Supplementary file 1 [file DataSheet3.zip › Data sheet 3/data sheet 3/Alix patients figure 4.TIF]

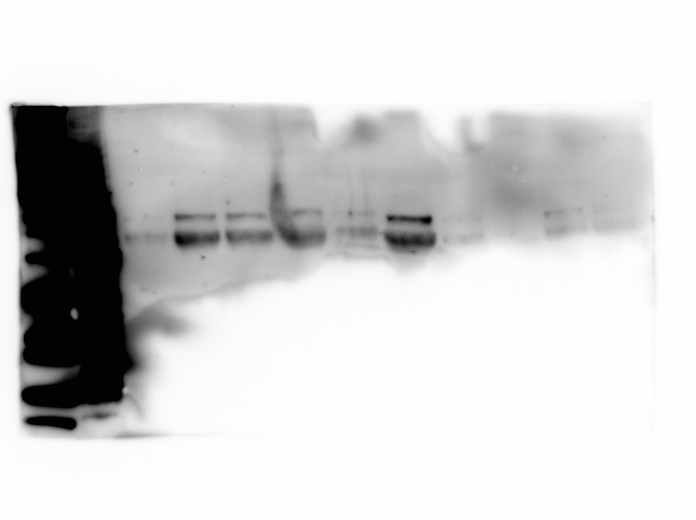

Supplement: Supplementary file 1 [file DataSheet3.zip › Data sheet 3/data sheet 3/alix_cells_fig 8.tif]

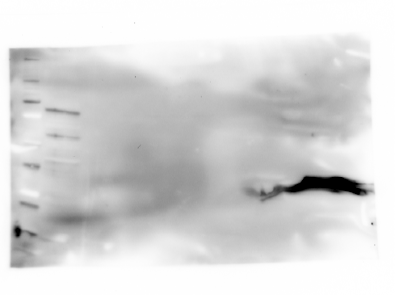

Supplement: Supplementary file 1 [file DataSheet3.zip › Data sheet 3/data sheet 3/Calnexin_cells_fig 8.tif]

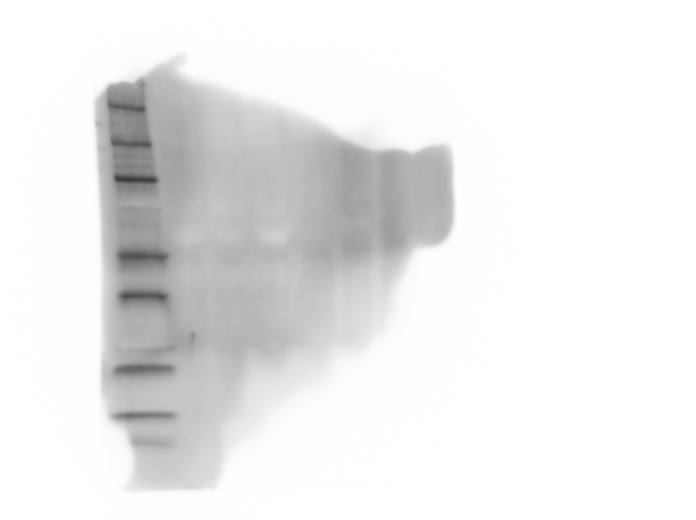

Supplement: Supplementary file 1 [file DataSheet3.zip › Data sheet 3/data sheet 3/Calnexin_patients_fig 4.tif]

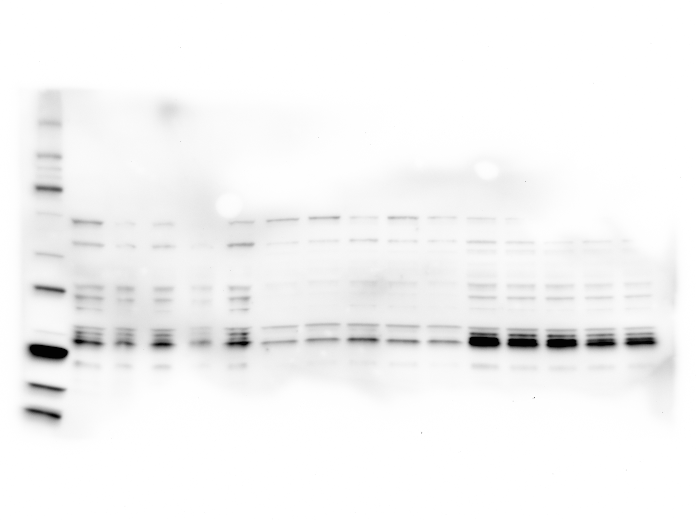

Supplement: Supplementary file 1 [file DataSheet3.zip › Data sheet 3/data sheet 3/CD81 cells+patients_fig 4-8.tif]

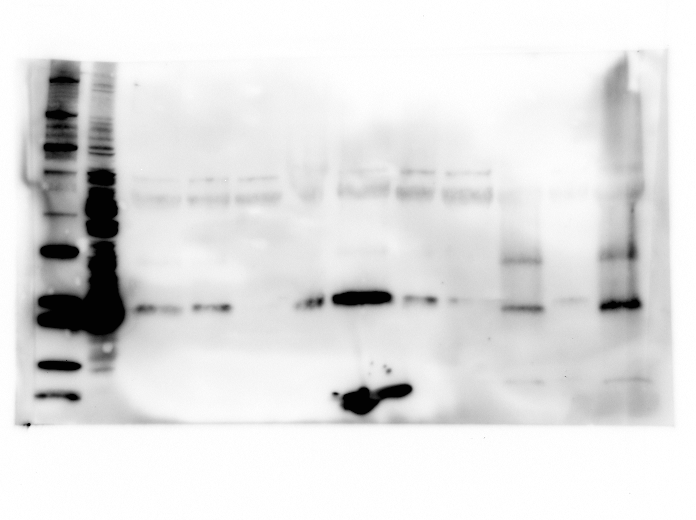

Supplement: Supplementary file 1 [file DataSheet3.zip › Data sheet 3/data sheet 3/CD9_cells_fig 8.tif]

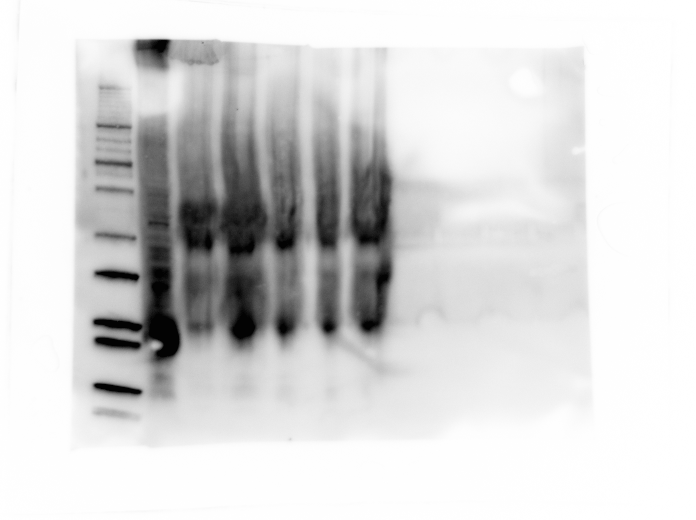

Supplement: Supplementary file 1 [file DataSheet3.zip › Data sheet 3/data sheet 3/CD9_patients_fig 4.tif]

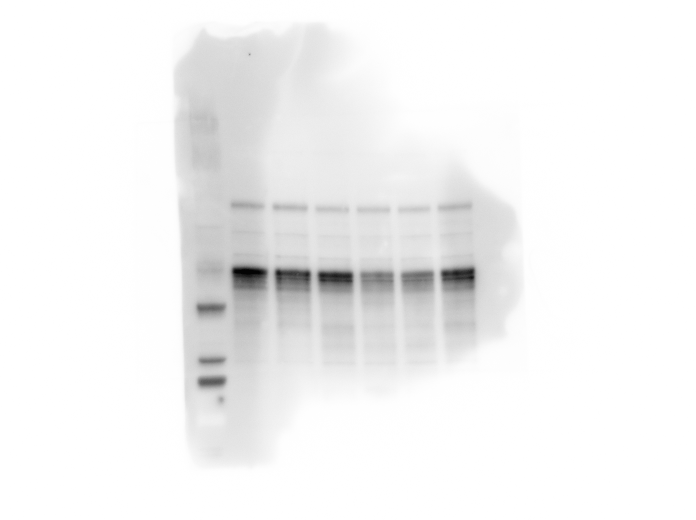

Supplement: Supplementary file 1 [file DataSheet3.zip › Data sheet 3/data sheet 3/TSG BC cells_fig 8.tif]

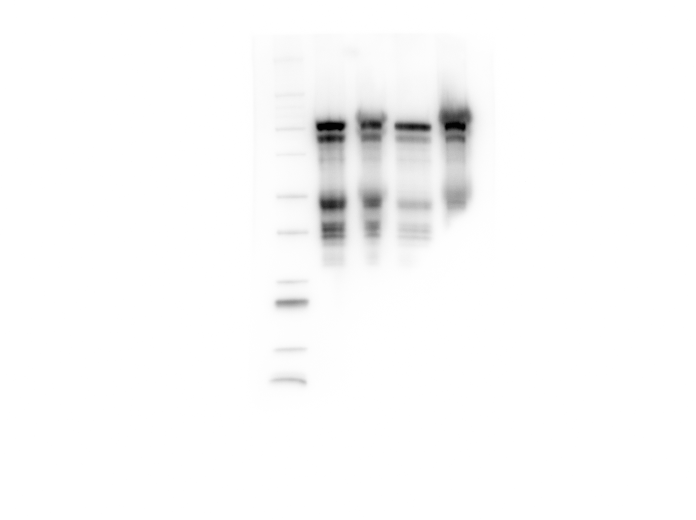

Supplement: Supplementary file 1 [file DataSheet3.zip › Data sheet 3/data sheet 3/TSG Huvec cells_fig 8.tif]

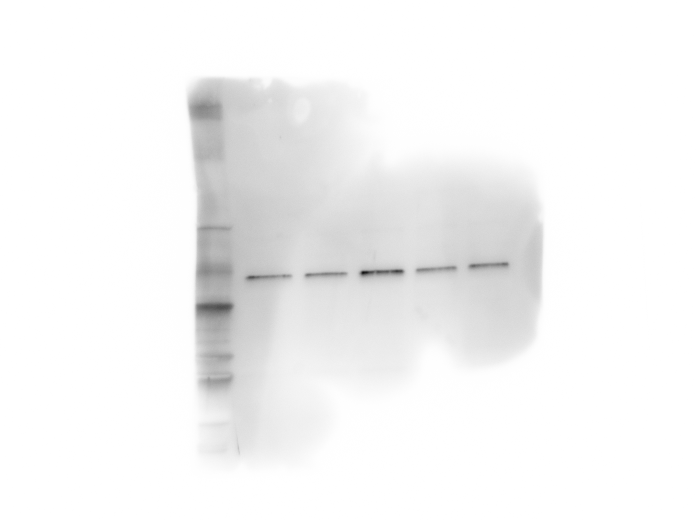

Supplement: Supplementary file 1 [file DataSheet3.zip › Data sheet 3/data sheet 3/TSG patients_fig 4.tif]

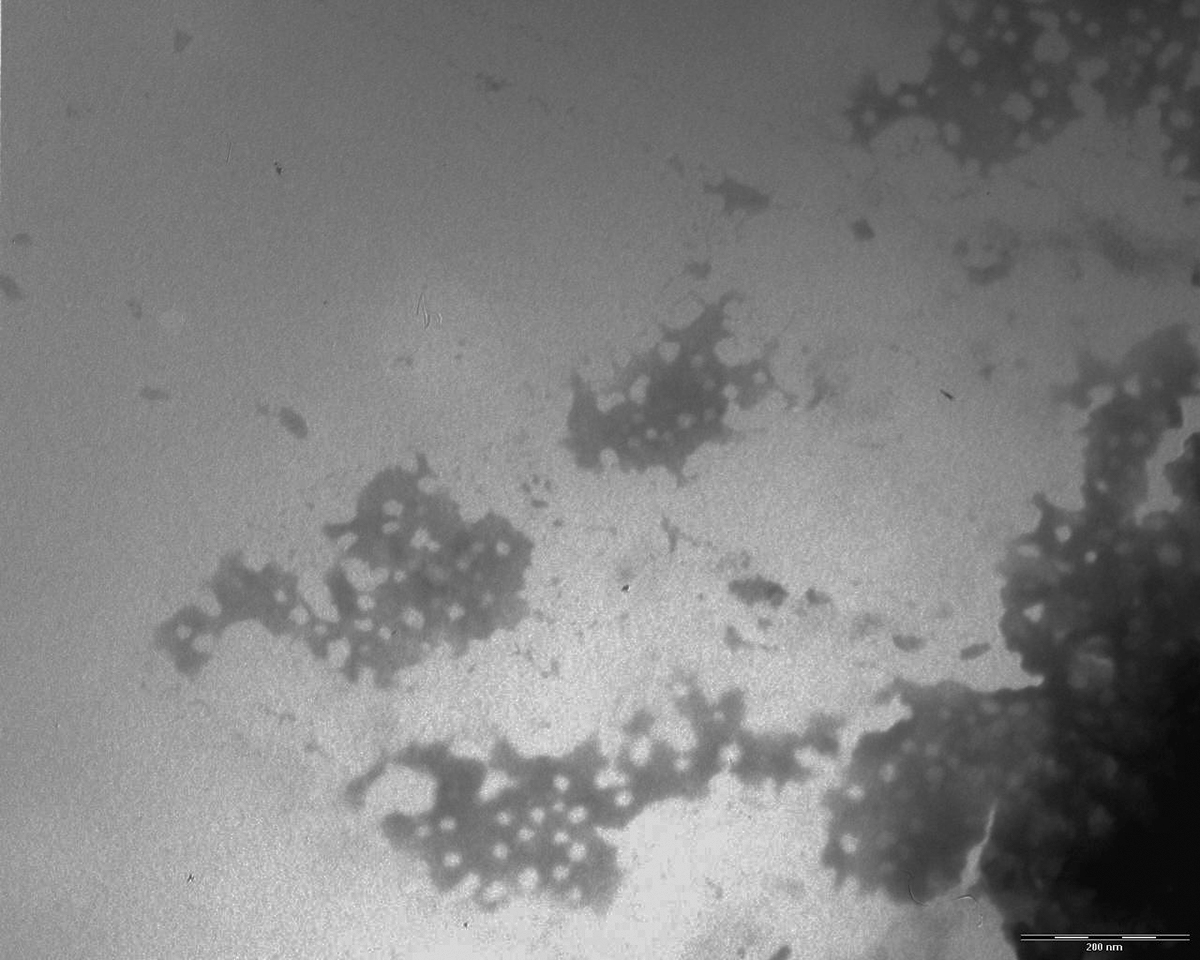

Supplement: Supplementary file 4 [file DataSheet2.zip › Data sheet 2/Huvec a.JPEG]

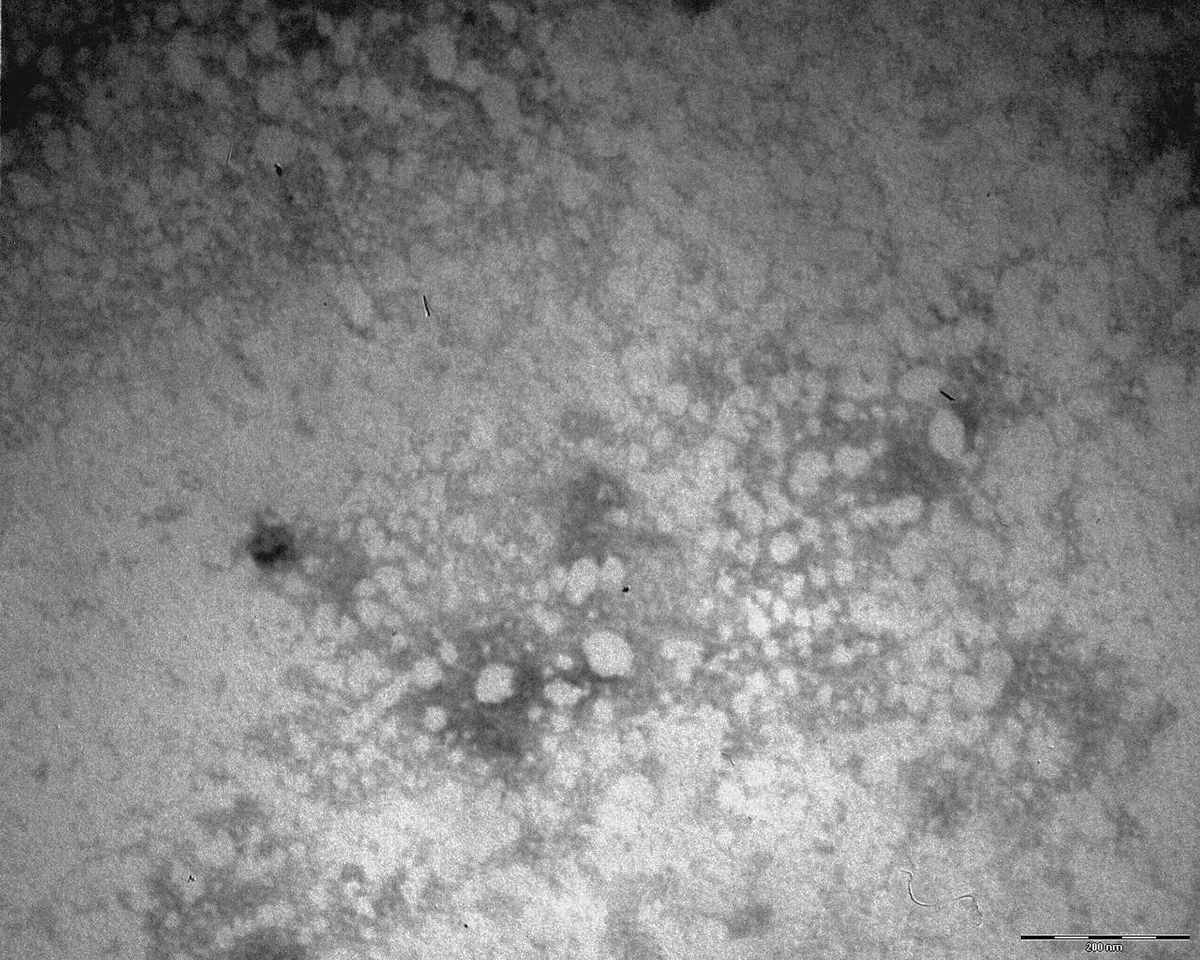

Supplement: Supplementary file 4 [file DataSheet2.zip › Data sheet 2/Huvec b.JPEG]

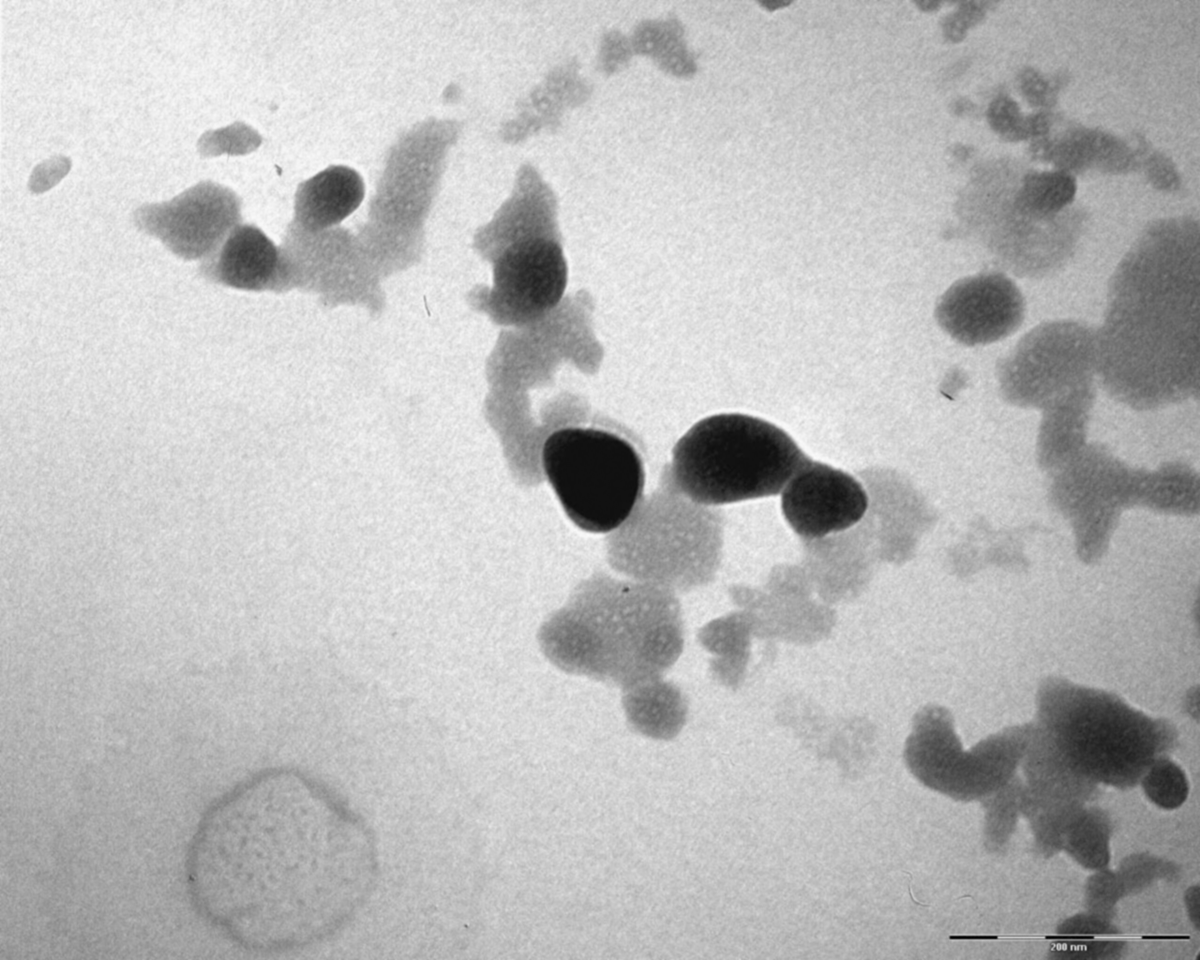

Supplement: Supplementary file 4 [file DataSheet2.zip › Data sheet 2/mcf7 a.tif]

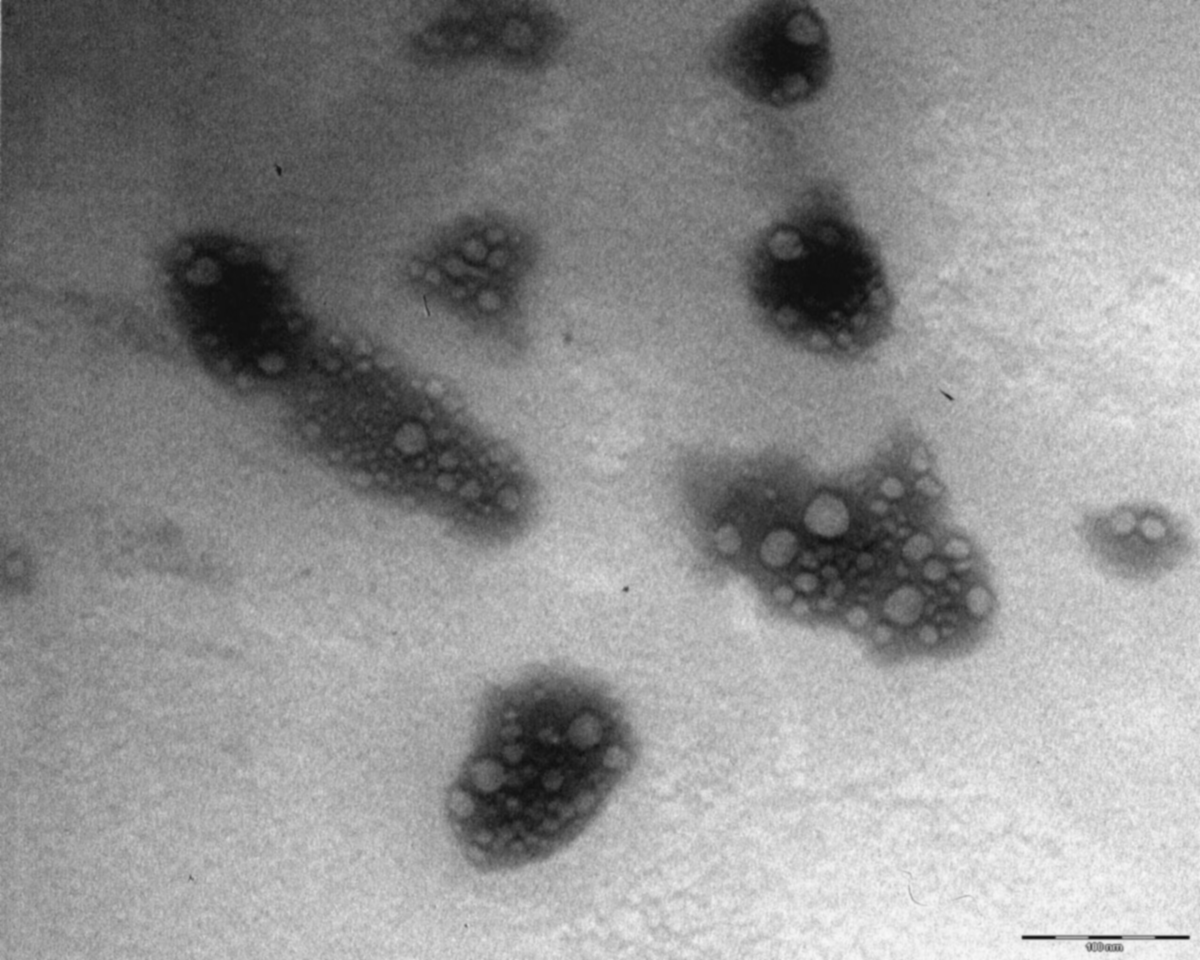

Supplement: Supplementary file 4 [file DataSheet2.zip › Data sheet 2/mcf7 b-c.tif]

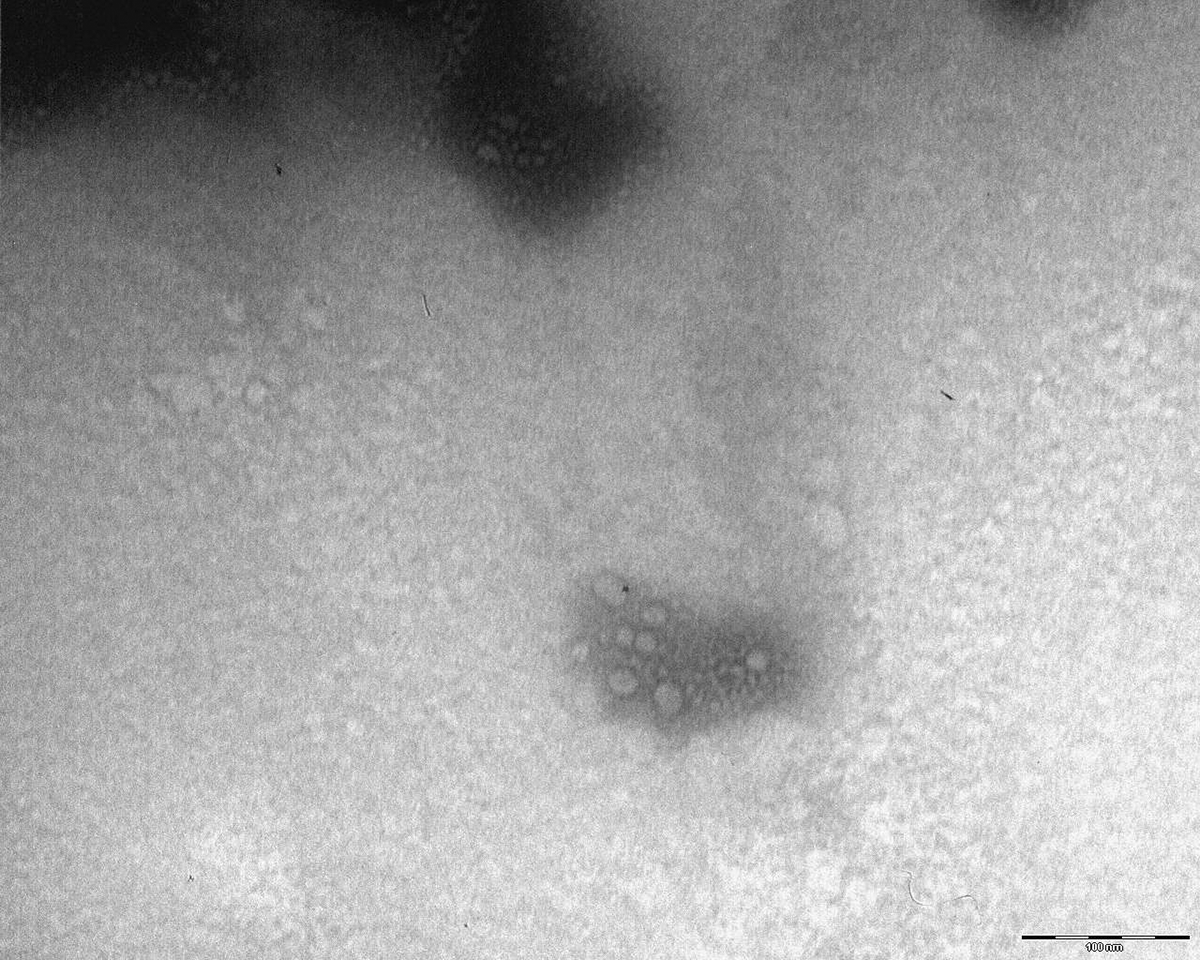

Supplement: Supplementary file 4 [file DataSheet2.zip › Data sheet 2/mcf7 d.tif]

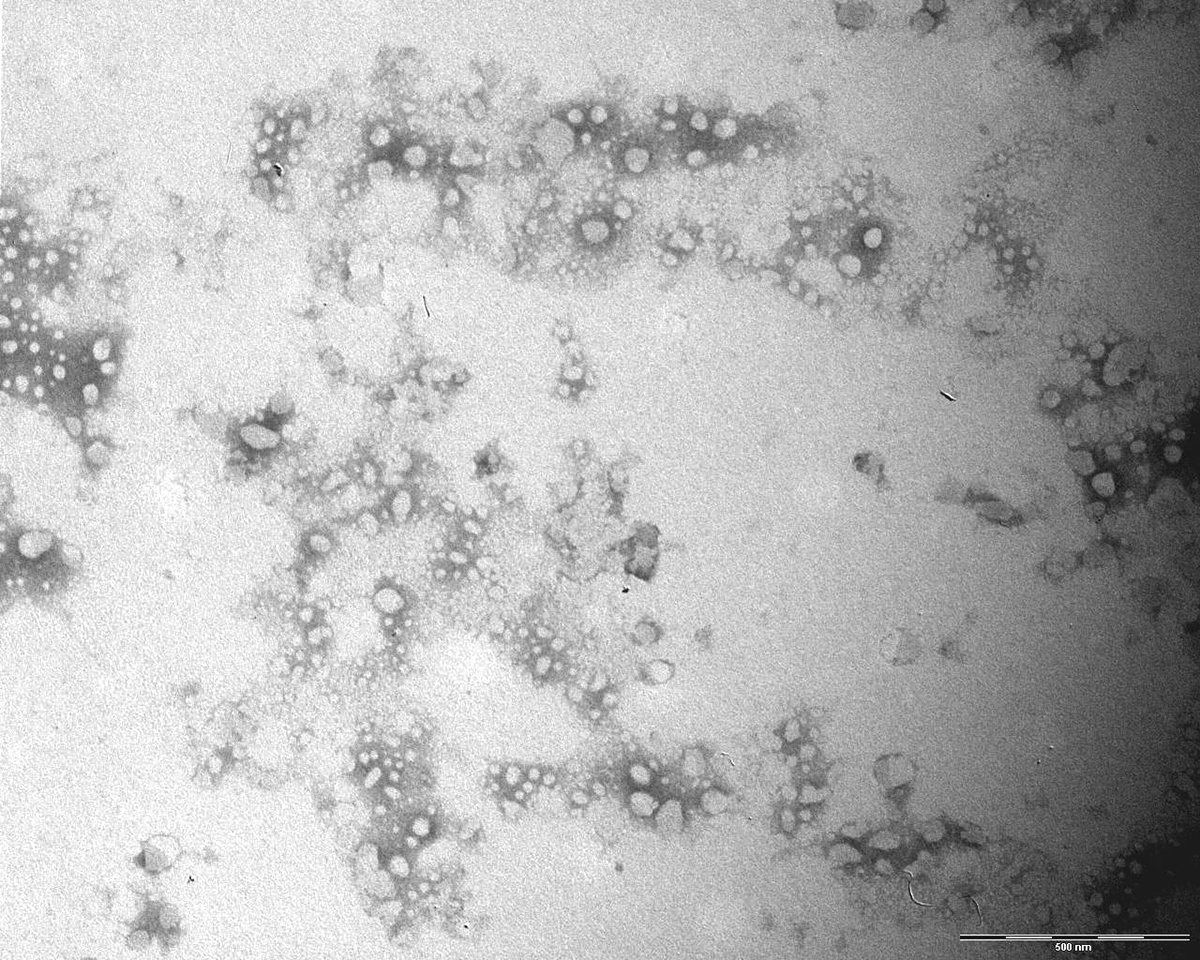

Supplement: Supplementary file 4 [file DataSheet2.zip › Data sheet 2/mda-a.tif]

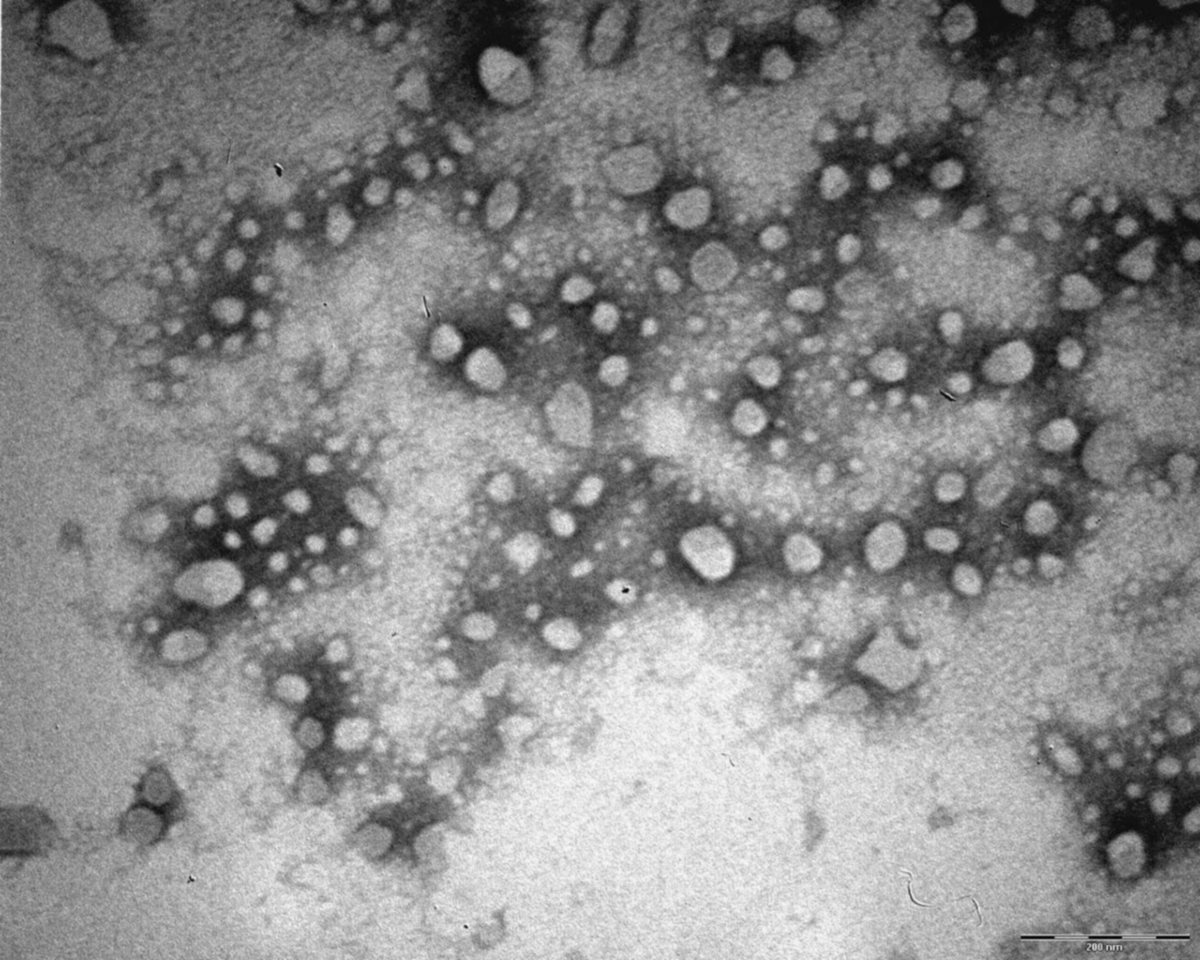

Supplement: Supplementary file 4 [file DataSheet2.zip › Data sheet 2/mda-b.tif]

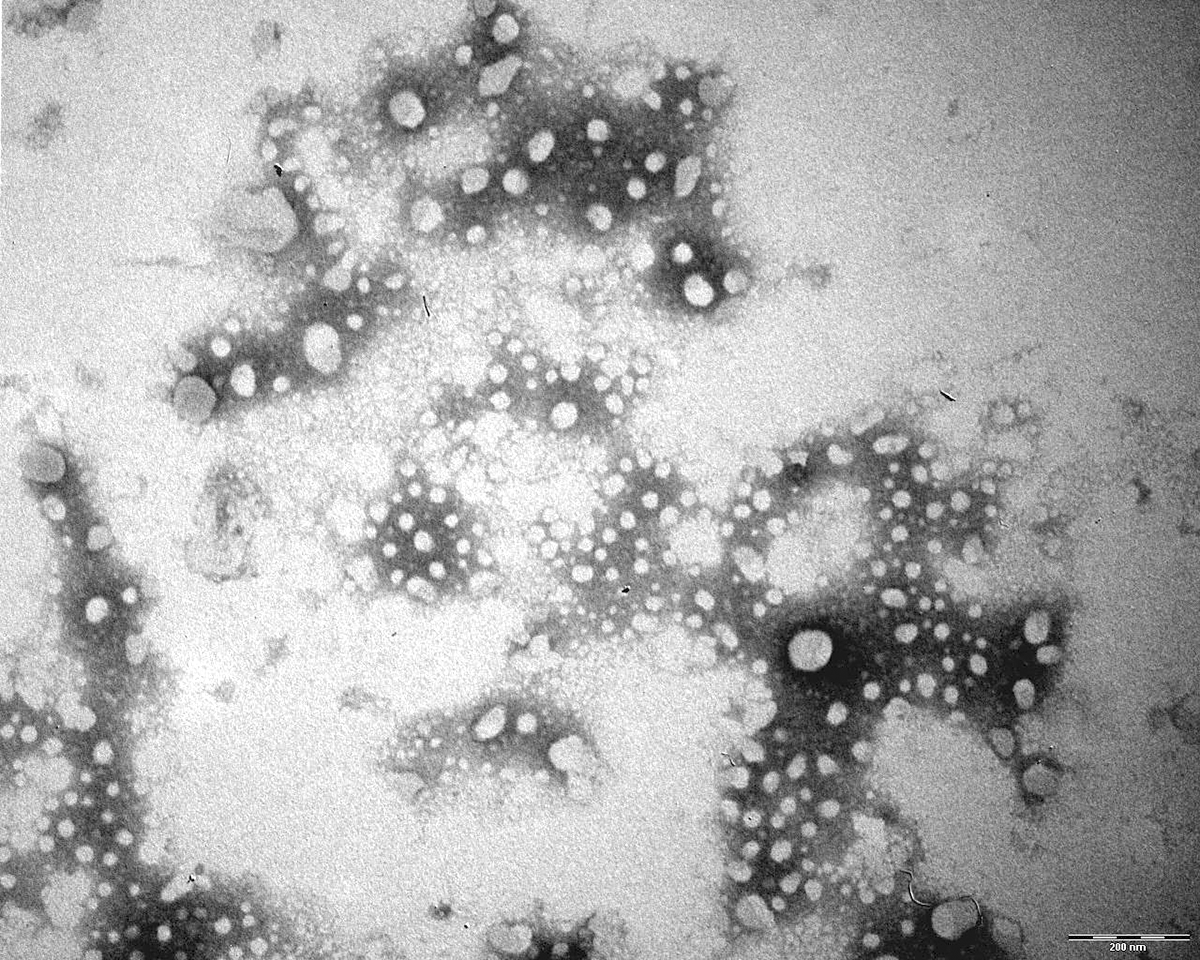

Supplement: Supplementary file 4 [file DataSheet2.zip › Data sheet 2/mda-c.tif]

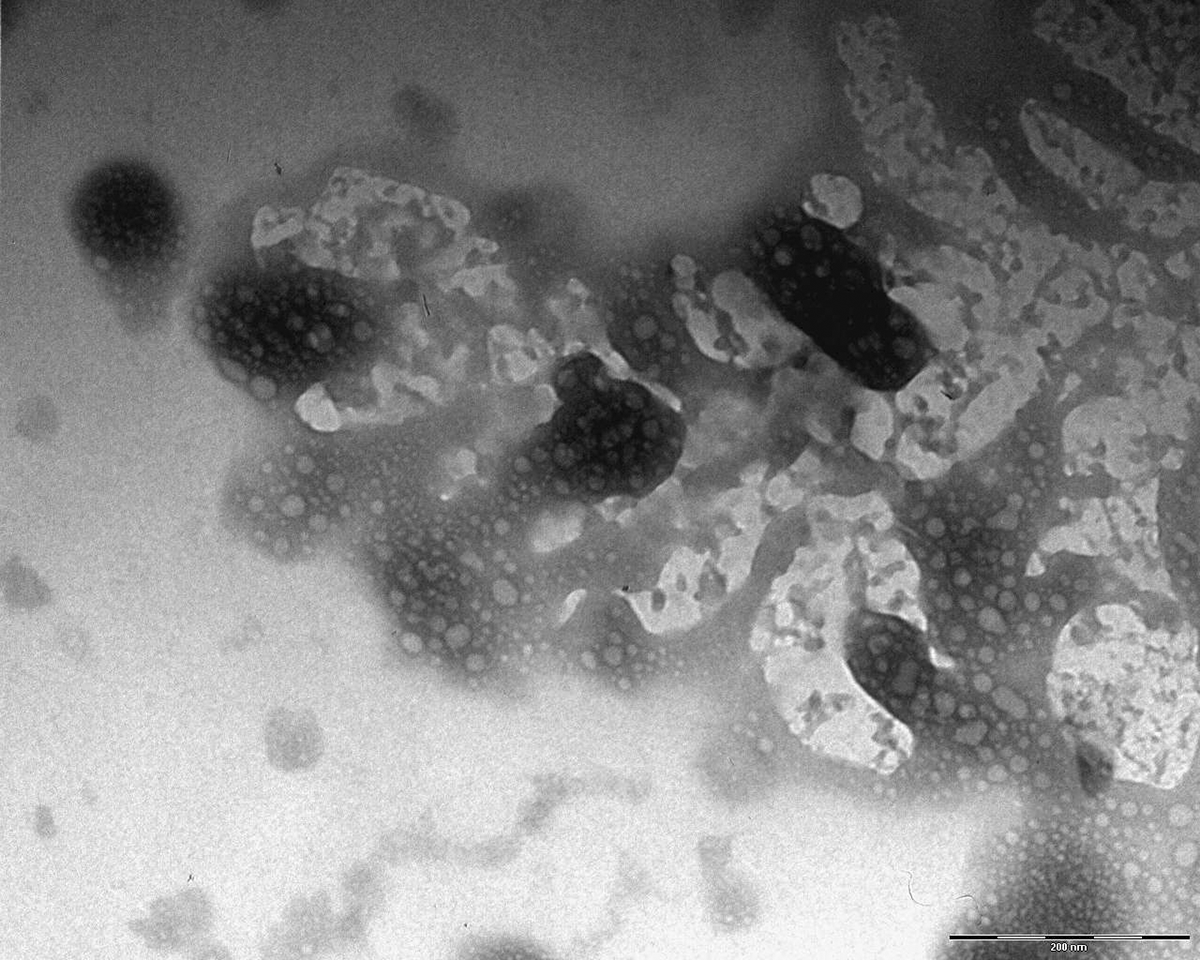

Supplement: Supplementary file 4 [file DataSheet2.zip › Data sheet 2/T47-d.tif]
