# Supplementary material for: Early Detection and Investigation of Extracellular Vesicles Biomarkers in Breast Cancer
Source: Front Mol Biosci. 2021 Nov 8;8:732900. doi: 10.3389/fmolb.2021.732900 (PMC8606536; doi:10.3389/fmolb.2021.732900)
Supplement: Supplementary file 2 [file DataSheet4.pdf]

SUPPLEMENTARY FIGURES

Uncropped western blotting images for figures 4 and 8

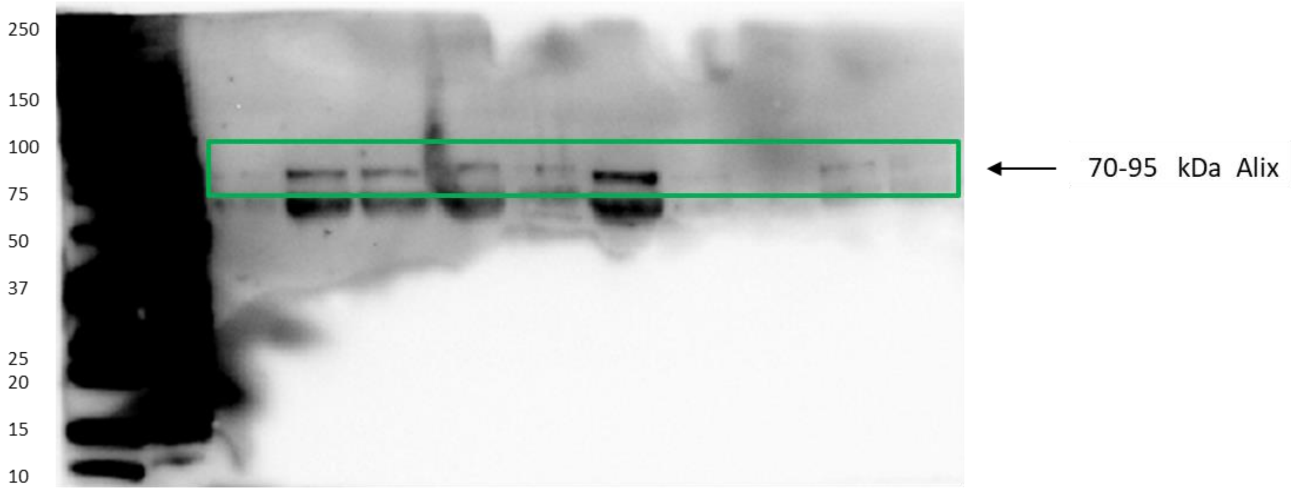

Alix in EVs from BC cells and Huvec (Fig. 8)

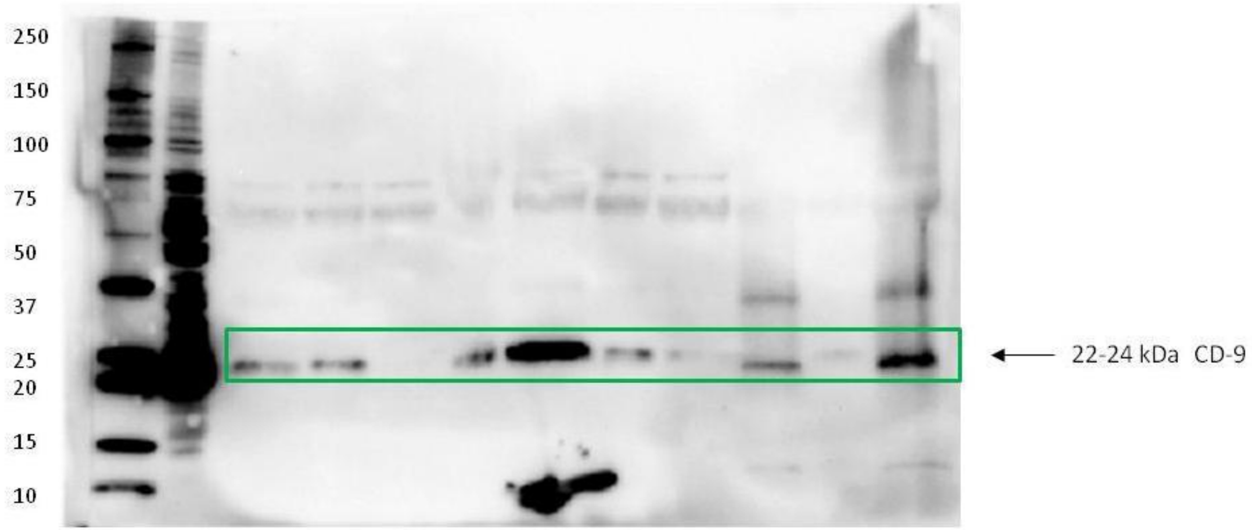

CD-9 in EVs from BC cells and Huvec (Fig.8)

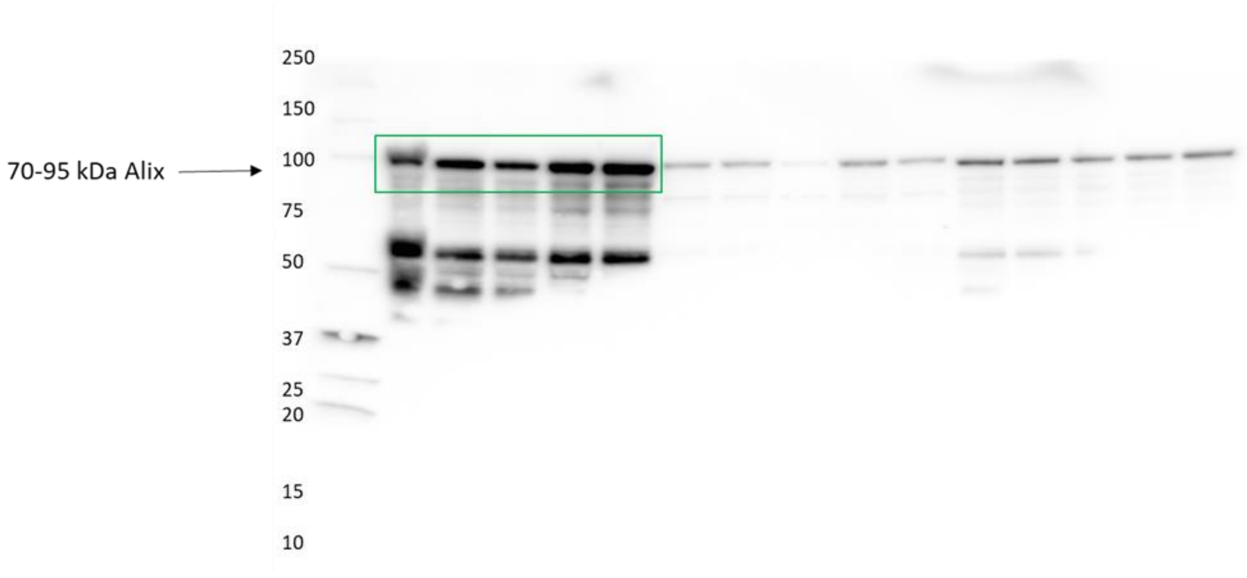

Alix in EVs derived from plasma of BC patients (Fig. 4)

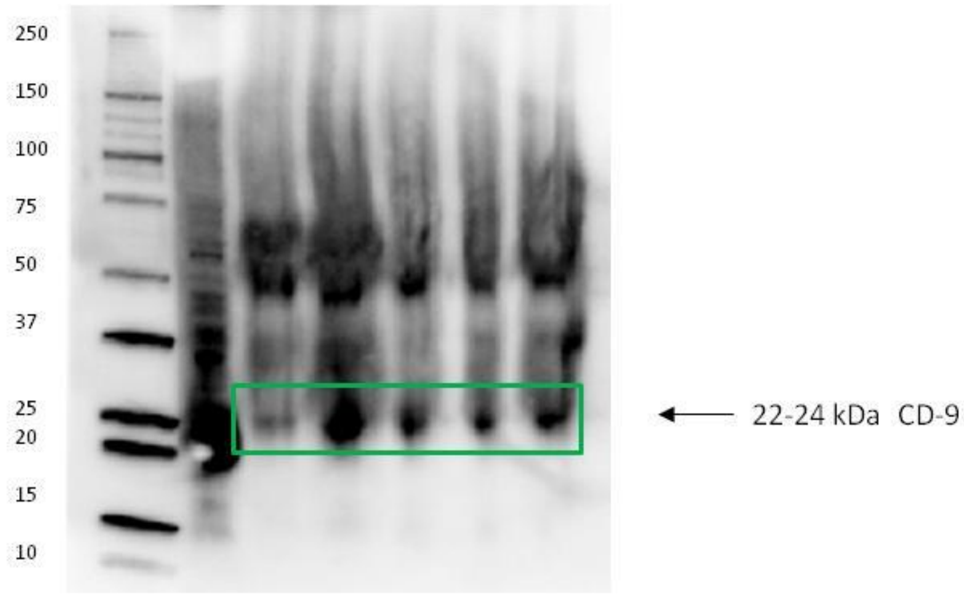

CD-9 in EVs derived from plasma of BC patients (Fig. 4)

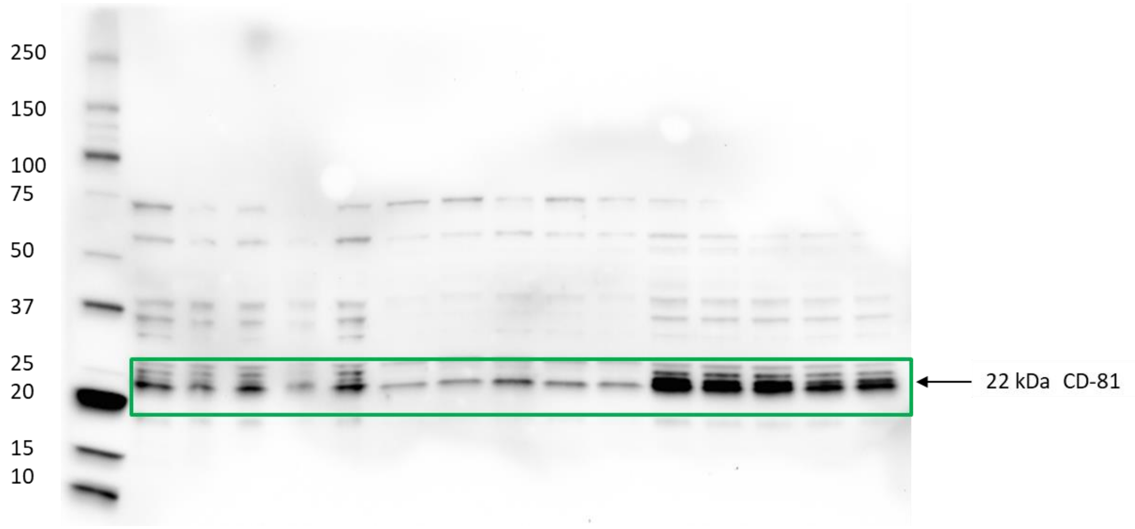

CD-81 in EVs derived from BC and Huvec cell lines (left) and BC patients (right) (Fig. 4 and 8)

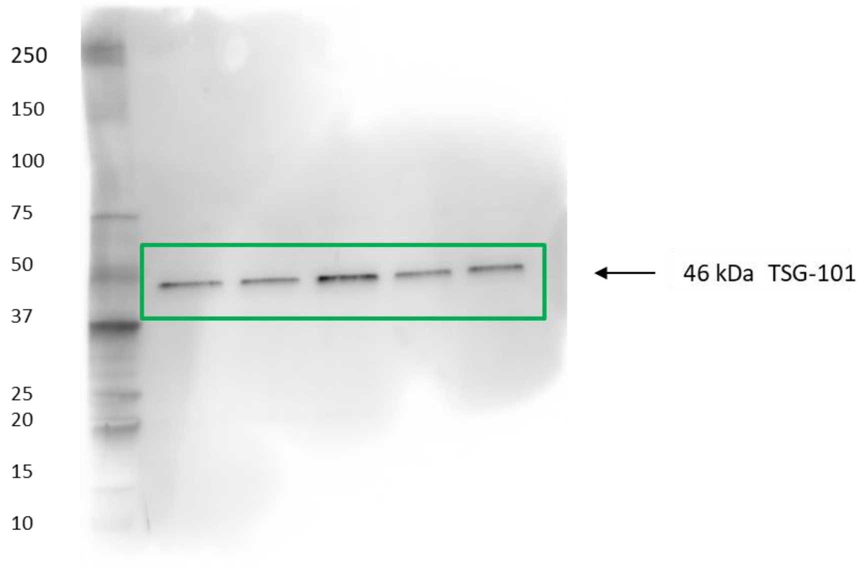

TSG-101 in EVs derived from plasma of BC patients (Fig. 4)

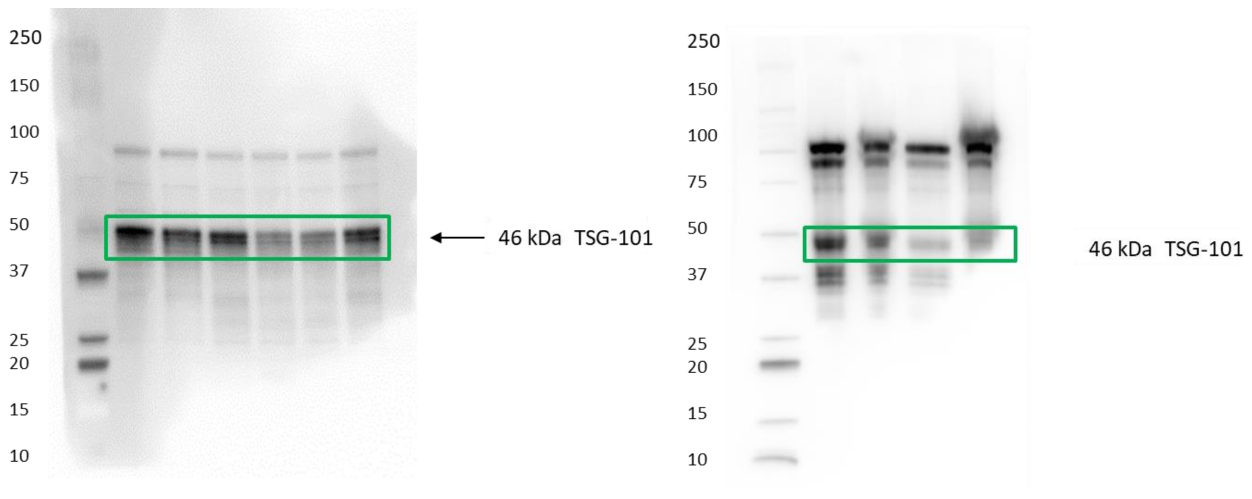

TSG-101 in EVs derived from BC cell lines (left) and Huvec cells (right) (Fig. 8)

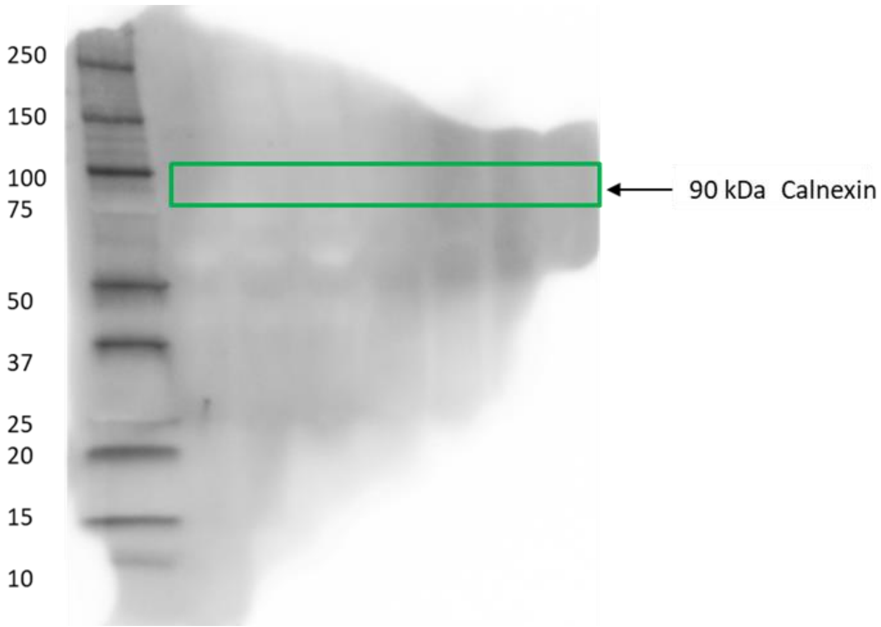

Calnexin in EVs derived from BC patients (Fig. 4)

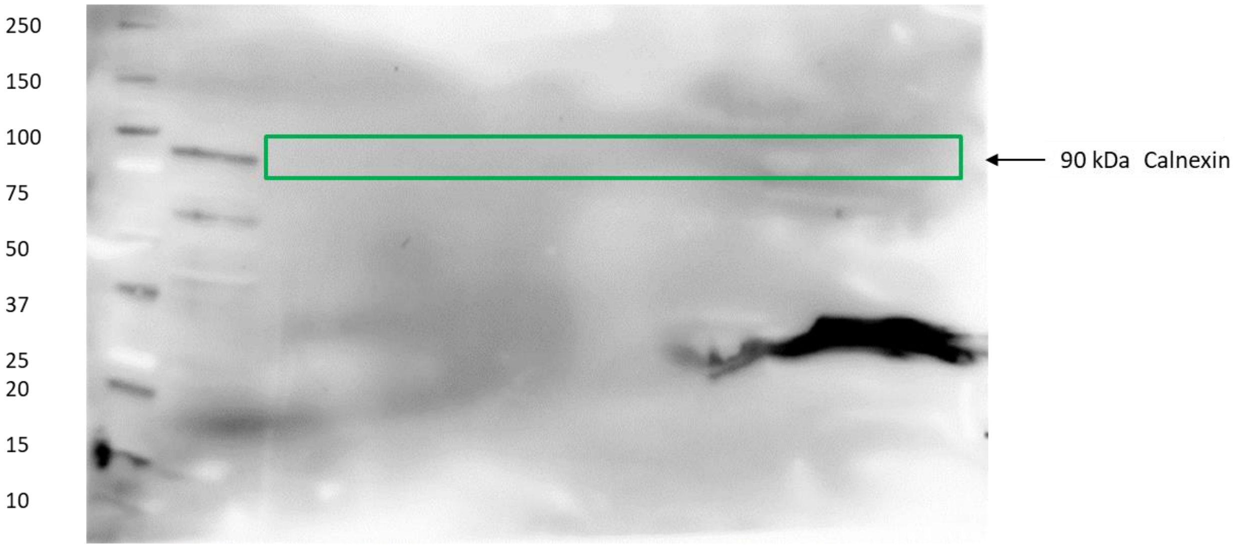

Calnexin in EVs derived from BC cells and Huvec (Fig. 8)
