# Supplementary material for: Early Detection and Investigation of Extracellular Vesicles Biomarkers in Breast Cancer
Source: Front Mol Biosci. 2021 Nov 8;8:732900. doi: 10.3389/fmolb.2021.732900 (PMC8606536; doi:10.3389/fmolb.2021.732900)
Supplement: Supplementary file 3 [file DataSheet1.pdf]

Supplementary Material

Supplementary Figure 1. Heatmap analysis of MFI of each plasma EVs marker within three time points. MFI: mean fluorescence intensity; EVs: extracellular vesicles

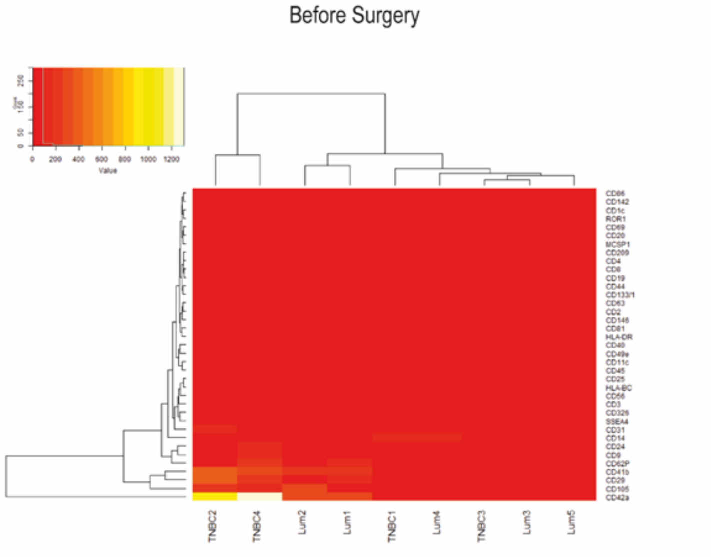
Before Surgery
Value
TNBC2
TNBC4
Lum2
Lum1
TNBC1
Lum4
TNBC3
Lum3
Lum5

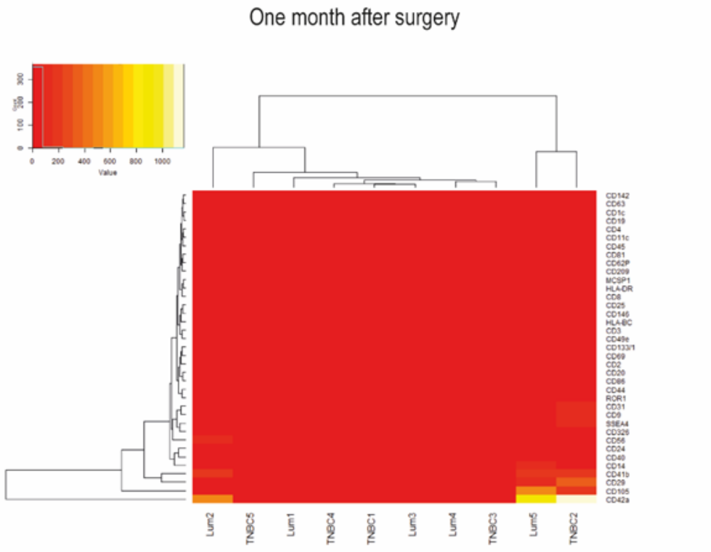
One month after surgery
Value
Lum2
TNBC5
Lum1
TNBC4
TNBC1
Lum3
Lum4
TNBC3
Lum5
TNBC2

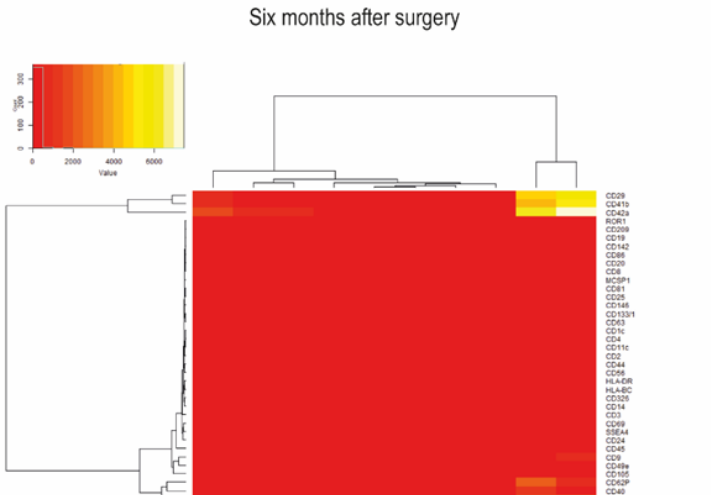
Six months after surgery
Value
